# The In Silico Prediction of Hotspot Residues that Contribute to the Structural Stability of Subunit Interfaces of a Picornavirus Capsid

**DOI:** 10.3390/v12040387

**Published:** 2020-03-31

**Authors:** Nicole Upfold, Caroline Ross, Özlem Tastan Bishop, Caroline Knox

**Affiliations:** 1 Department of Biochemistry and Microbiology, Rhodes University, Grahamstown 6140, South Africa; caroline.knox@ru.ac.za; 2 Research Unit in Bioinformatics (RUBi), Department of Biochemistry and Microbiology, Rhodes University, Grahamstown 6140, South Africa; caroross299@gmail.com (C.R.); O.TastanBishop@ru.ac.za (Ö.T.B.)

**Keywords:** assembly, axis of symmetry, capsid, cardiovirus, hotspot, pentamer, protomer, protein–protein interaction

## Abstract

The assembly of picornavirus capsids proceeds through the stepwise oligomerization of capsid protein subunits and depends on interactions between critical residues known as hotspots. Few studies have described the identification of hotspot residues at the protein subunit interfaces of the picornavirus capsid, some of which could represent novel drug targets. Using a combination of accessible web servers for hotspot prediction, we performed a comprehensive bioinformatic analysis of the hotspot residues at the intraprotomer, interprotomer and interpentamer interfaces of the Theiler’s murine encephalomyelitis virus (TMEV) capsid. Significantly, many of the predicted hotspot residues were found to be conserved in representative viruses from different genera, suggesting that the molecular determinants of capsid assembly are conserved across the family. The analysis presented here can be applied to any icosahedral structure and provides a platform for in vitro mutagenesis studies to further investigate the significance of these hotspots in critical stages of the virus life cycle with a view to identify potential targets for antiviral drug design.

## 1. Introduction

The *Picornaviridae* are a heterogeneous family of small RNA viruses that includes etiological agents of significant human and animal diseases [1], such as foot-and-mouth disease virus (FMDV), poliovirus (PV), enterovirus 71 (EV-71) and hepatitis A virus (HAV) [2]. Despite their clinical and economic significance, no antiviral therapy is commercially available for the treatment of picornavirus infections [3,4] and effective vaccines are only available for PV, HAV and FMDV [5,6,7].

Picornavirus capsids are nonenveloped icosahedral multimers, comprising 60 copies of four capsid proteins (VP1-4), that are assembled through the consecutive oligomerisation of polypeptide subunits in a stepwise manner. Following the cleavage of the viral polyprotein, VP0, VP1 and VP3 immediately form the protomer. Five protomers subsequently assemble into the pentameric subunit, and twelve pentamers combine to yield the full capsid. A final cleavage event results in maturation of the capsid and separation of VP0 into VP2 and 4, and the resulting capsids have two-fold, three-fold and five-fold axes of symmetry [8]. During the capsid assembly cascade, a network of noncovalent interactions is formed between the capsid protein precursors, which are essential for the self-assembly, structural integrity and stability of the capsid [9,10]. They must be strong enough to prevent capsid dissociation in the harsh extracellular environment yet remain labile to allow uncoating and genome release inside the cell [10,11]. Importantly, interface residues do not equally participate in the binding of protein complexes. Rather, a small subset of residues termed “hotspots” contributes significantly to the binding energy, specificity and stability of protein–protein associations [12,13].

Considering the importance of intersubunit hotspot residues, and the interactions they form, to capsid assembly and stability [14], it has been realised that they provide attractive targets for the rational design of capsid-specific antivirals or could be manipulated to yield virus particles with improved stability for use in biotechnology and medicine [15,16]. Thus, studies have attempted to identify these important residues and determine their role in capsid assembly and viral function [17,18,19]. Most of these studies have relied upon the systematic dissection of individual interfacial residues by in vitro mutagenesis [9,11,20]. Such an approach is significantly challenging when large interfaces, like those found in viral capsids, are to be analysed. Consequently, authors have attempted to theoretically assess residues at capsid subunit interfaces and generate a map of specific residues for further experimental analysis using computational methods such as electrostatic energy calculations [21] and free energy functions [22].

Tools for the prediction of hotspots at protein–protein interfaces have been developed using a variety of models and approaches. Energy-based models, such as computational alanine scanning, use free energy functions to estimate the change in binding energy (ΔΔG binding) between the wild type and mutant protein complex upon mutation of individual amino acid residues to alanine [23,24]. Drawbacks to this method are firstly, that residues which form contacts through main-chain and not side-chain atoms are ignored and secondly, protein conformations may be altered or destabilised during the alanine substitution, leading to an increase in false positives [25,26]. Molecular dynamics (MD) simulations also estimate the free energy of association and have excellent predictive power but are not suited to studies of extensive interfaces because of their computational cost and difficulty of execution [27,28,29]. Feature-based approaches use machine learning models to evaluate several sequence and structural characteristics of interfaces for hotspot prediction such as residue location, type, conservation and solvent accessibility [30] and are computationally efficient, but may be oversensitive to the selected features they consider [31]. Several studies have demonstrated that hotspot prediction can be improved by combining various energetic- and feature-based models [32,33,34,35].

Few studies have attempted to theoretically investigate the residues critical to capsid assembly and stability in viruses within the *Picornaviridae*. We previously developed an in silico approach employing computational alanine scanning to identify a network of hotspot residues in conserved motifs within the intraprotomer, interprotomer and interpentamer subunit interfaces of enterovirus capsids that are possibly involved in capsid uncoating and RNA release [36]. The study contributed to the understanding of the conserved molecular determinants that modulate enterovirus capsid stability at a genus level; however, knowledge regarding the residues that critically contribute to the assembly and stability of capsids belonging to picornaviruses from other genera remains limited. Identifying these specific residues in other picornaviruses is imperative for a comprehensive understanding of picornavirus capsids and how they are assembled and disassembled, but also for the development of improved broad-spectrum strategies to control these significant viruses. The present study elucidates the residues at the intraprotomer, interprotomer and interpentamer interfaces that contribute to the stability and assembly of the Theiler’s murine encephalomyelitis virus (TMEV) capsid, using an in silico screen that combines five readily accessible energy- and feature-based models for hotspot prediction. The hotspot residues are further assessed to differentiate between those which are unique to the TMEV capsid and those which are conserved within capsids of viruses across the family. Our findings demonstrate that hotspot residues unique to TMEV are predominantly found at the intra- and interprotomer interfaces of the capsid, but many hotspots in the same interfaces and particularly those between pentamers are conserved in viruses from other genera, suggesting that molecular determinants of capsid stability may be somewhat conserved across the family.

## 2. Materials and Methods

**Figure 1:**
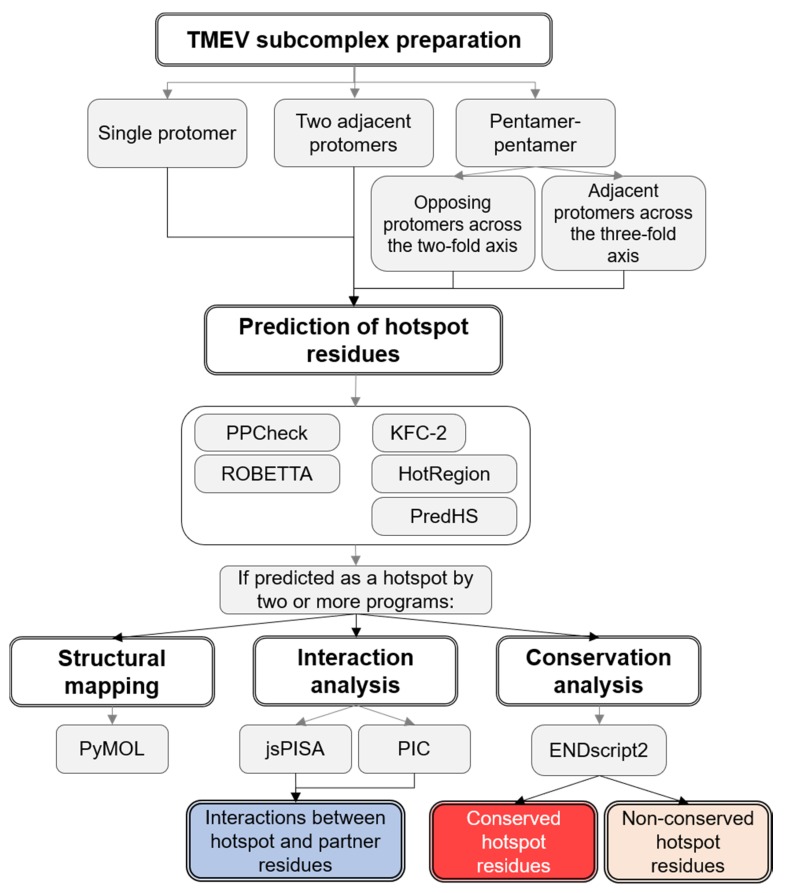
Flow diagram of the methodology used in the study. Theiler’s murine encephalomyelitis virus (TMEV) subcomplexes representing the different subunit interfaces of TMEV were extracted from the homology model of the TMEV GDVII capsid generated previously [37,38]. The complexes were submitted to five tools for hotspot prediction. The prediction of protein interactions was performed using PIC and jsPISA. Hotspot residues and their interacting partners were mapped to the capsid complexes using PyMOL. Finally, ENDscript2 was used to assess the conservation of individual hotspot residues across the picornavirus family.

An overview of the methodology used to identify hotspot residues within the intraprotomer, interprotomer and interpentamer interfaces of TMEV is provided in Figure 1.

### 2.1. Preparation of TMEV Subcomplexes

**Figure 2:**
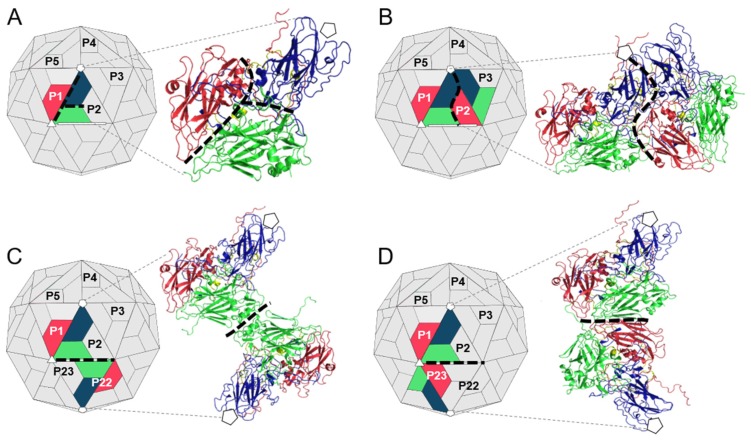
TMEV GDVII capsid subcomplexes for subunit interface hotspot and residue–residue interaction prediction. The respective subcomplexes were extracted from the biological assembly of TMEV GDVII in PyMOL. (**A**) The intraprotomer complex, consisting of a single protomer of the capsid. (**B**) The interprotomer complex, comprising two adjacent protomers within a pentamer. (**C**) The first complex of the interpentamer interface, consisting of two opposing protomers from two adjacent pentamers. (**D**) The second complex of the interpentamer interface, consisting of two adjacent protomers from two adjacent pentamers. Capsid proteins: VP1 (blue); VP2 (green); VP3 (red); and VP4 (yellow). The intraprotomer, interprotomer and interpentamer interfaces are denoted by black dashed lines. The five-fold, three-fold and two-fold axes are indicated by white pentagons, triangles and rectangles, respectively.

Due to icosahedral symmetry, the protomer subunits and contacts between them are repeated 60 times across the picornavirus capsid. To reduce computing time, single complexes representing the intraprotomer, interprotomer and interpentamer interfaces were generated by extracting the relevant protomer subunits from a homology model of the complete biological assembly of TMEV GDVII [37,38]. Protomers in the biological assembly are numbered P1–P60 according to their position in the capsid, where P1–P5 constitutes the first pentamer and P6–P10 the second pentamer. Protomers in the derived subcomplexes were labelled according to this scheme. To represent the intraprotomer interface, a single TMEV protomer (P1) was extracted (Figure 2A). To represent the interprotomer interface, two adjacent protomers within a single pentamer were extracted (P1 and P2) (Figure 2B). Finally, to represent the interpentamer interface, two complexes were generated using three protomers. The first complex, shown in Figure 2C, consisted of a protomer (P1) from one pentamer and an opposing protomer from the adjacent pentamer located across the two-fold axis (P22). The second complex comprised the same protomer (P1), but the second protomer was the adjacent protomer (P23) in the adjacent pentamer located next to the three-fold axis of symmetry (Figure 2D).

### 2.2. Hotspot Prediction

A combination of five in silico tools was implemented for the prediction of hotspot residues within the TMEV capsid. These prediction programs are included in Table 1. The files of the TMEV intraprotomer, interprotomer and interpentamer (A and B) subcomplexes (Figure 2) were individually submitted to each web server for hotspot residue analysis. A residue was only considered as a hotspot if it was identified by at least two of the five prediction methods. The results were mapped to the crystal structures of the individual TMEV subcomplexes for visualization in PyMOL [39].

### 2.3. Prediction of Interacting Residues

The intraprotomer, interprotomer and interpentamer complexes were individually submitted to the Protein Interactions Calculator (PIC) [45] and the Protein Interactions, Surfaces and Assemblies (jsPISA) web server [46] to calculate protein–protein interactions (Table 2). The results generated by the two web servers were then used to generate interacting network diagrams of the hotspots and their interacting partners.

### 2.4. Residue Conservation

Hotspot residue conservation was assessed by analysing the sequence and structural conservation of each capsid protein using ENDscript2 (http://endscript.ibcp.fr), which identifies and aligns homologous sequences in Clustal Omega, then adds protein secondary structure information from corresponding PDB files [47,48]. The single TMEV protomer complex was submitted to the server, and individual protein chains A, B, C and D representing VP1-4, respectively, were analysed separately. ENDscript2 generated sequence-based alignments without secondary structure information for VP1 and VP4 as few homologous structures were available.

## 3. Results

### 3.1. Analysis of the Residues that Contribute to the Intraprotomer Interfaces of TMEV GDVII

#### 3.1.1. Predicted Hotspots at the Intraprotomer Interfaces

**Figure 3:**
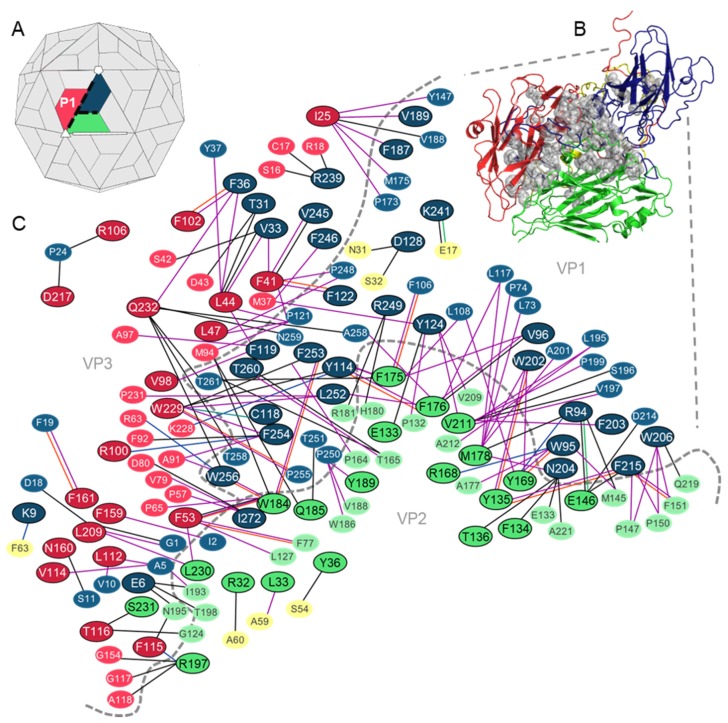
Hotspot residues and their interacting partners at the intraprotomer interfaces of the TMEV capsid. (**A**) Structure of the picornavirus capsid showing a single protomer. The intraprotomer interfaces between the three main capsid proteins are indicated by dashed black lines. (**B**) Structural cartoon mapping of predicted hotspot residues (shown as grey spheres) at the intraprotomer interfaces. (**C**) Schematic network of hotspot residues and the contacts made with their residue binding partners at the intraprotomer interfaces. Hotspot residues are outlined in black. Residues without outlines are non-hotspot partner residues. VP1 residues (blue); VP2 residues (green); VP3 residues (red); VP4 residues (yellow). Noncovalent interactions between hotspots and partners are shown as solid lines: hydrogen bonds (black lines); hydrophobic (purple lines); aromatic (orange lines); aromatic–sulphur (turquoise lines); cation–pi (blue lines); ionic (green lines) interactions.

Seventy-two residues were identified as hotspots at the interfaces of the protomer complex (Figure 3), by at least two of the prediction web servers (Table S1). Thirty-two of these residues belong to VP1 and the remaining 40 hotspots are split equally between VP2 and VP3 (Figure 3B,C).

Protein interaction analysis confirmed that all predicted hotspots are involved in at least one interaction across the intraprotomer interface. Most hotspots were found to form contacts with residues across a single protein–protein interface; however, residue Q185 (VP2) was predicted to form hydrophobic interactions with residues T251 (VP1) and M94 (VP3) and contribute to the stability of both protein–protein interfaces. The analysis also revealed that of the 72 hotspot residues, 44 form interactions with other hotspot residues and 39 form multiple contacts. Both hydrogen bonds and hydrophobic interactions were frequent at the VP1–VP3 and VP2–VP3 interfaces, while the contacts between hotspots and their interacting partners at the VP1–VP2 interface were predominantly hydrophobic (Figure 3C).

Several of the intraprotomer hotspots within the TMEV capsid were previously found to be functionally important. Roles for these residues in capsid stability and receptor binding were previously documented and are summarized in Table 3.

#### 3.1.2. Intraprotomer Hotspot Residues in TMEV that are Conserved with Residues in Other Viruses of the Family

**Figure 4:**
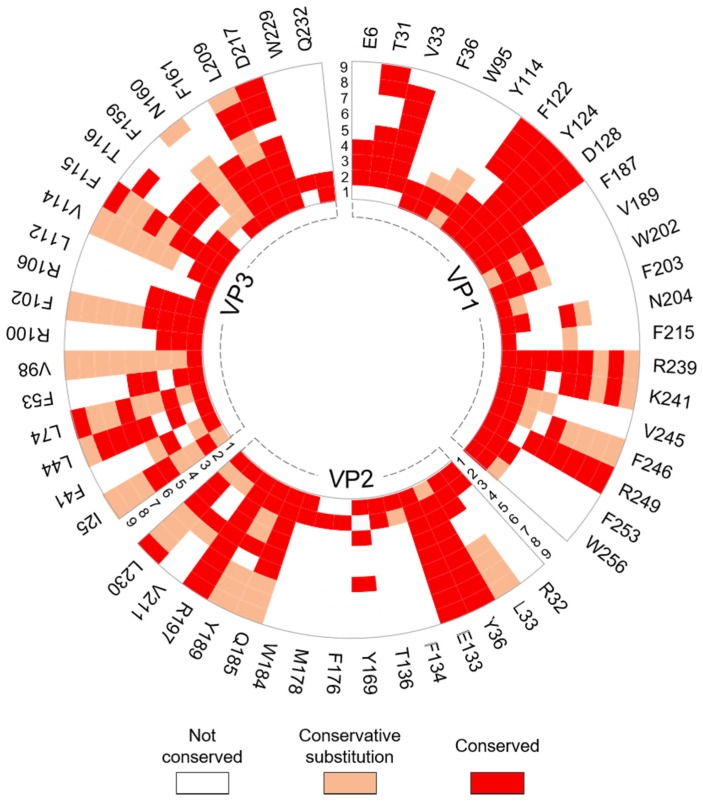
Radial map of the conservation of TMEV intraprotomer residues identified as hotspots across respective picornavirus species. The individual TMEV capsid proteins were submitted to ENDScript2 for conservation analysis. The degree of conservation for each TMEV hotspot was compared in nine representative picornaviruses across four genera. Identity of viruses: 1) *Cardiovirus B* (SAFV; PDB: 5CFC/5A8F); 2) *Cardiovirus A* (EMCV; PDB: 2MEV); 3) *Senecavirus* (SVV-1; PDB: 3CJI); 4) *Equine Rhinitis A Virus* (ERAV; PDB: 2WFF); 5) *Foot-and-mouth disease virus* (FMDV; PDB: 5ACA); 6) *Enterovirus A* (EV-71; PDB: 3VBS); 7) *Enterovirus B* (CAV-9; PDB: 1D4M); 8) *Rhinovirus A* (HRV-3; PDB: 1RHI); 9) *Rhinovirus B* (HRV-2; PDB: 1FPN). Full alignments are available in Supplementary Figures S1–S4.

Sequence and structural alignments using ENDscript2 revealed that 57 intraprotomer hotspots are conserved with residues in the capsid proteins of other viruses in the family. Hotspots Y124, D128 and R239 in VP1, Y36, E133, Q185, R197 and L230 in VP2 and I25, L47, V98, F102, F115 and L209 in VP3 were either universally conserved or were subject to conservative mutation in representatives from the cardio-, seneca-, aphtho- and enteroviruses (Figure 4). Only five of the hotspot residues, Y124, N204, K241, F246 and R249, all belonging to VP1, are conserved with residues in the *Enterovirus* and *Parechovirus* genera that were previously reported or suggested to be critical for virus growth, capsid stability and protein–RNA interactions. These hotspots and their corresponding residues in other viruses are summarized in Table 4.

### 3.2. Analysis of the Residues that Contribute to the Interprotomer Interfaces of TMEV GDVII

#### 3.2.1. Predicted Hotspot Residues at the Interprotomer Interfaces

**Figure 5:**
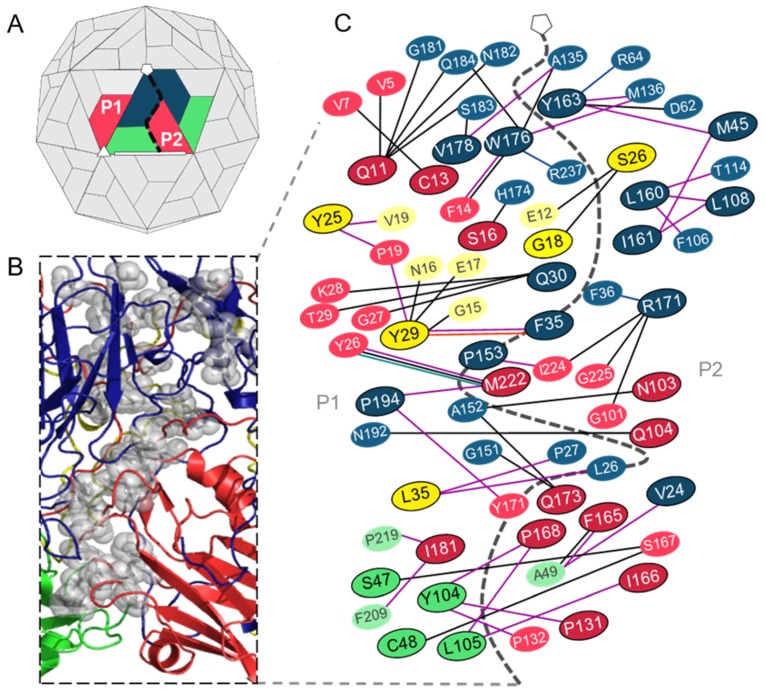
Hotspot residues and their interacting partners at the interprotomer interfaces of the TMEV capsid. (**A**) Structure of the picornavirus capsid showing two protomers within a pentamer that form the interprotomer interface (dashed black line). (**B**) Structural cartoon mapping of predicted hotspot residues (shown as grey spheres) at the interprotomer interfaces. (**C**) Network of hotspot residues and the contacts made with their residue binding partners at the interprotomer interface. Hotspot residues are outlined in black; partner non-hotspots are not outlined. VP1 residues (blue); VP2 residues (green); VP3 residues (red); VP4 residues (yellow). Noncovalent interactions between hotspots and partners are shown as solid lines: hydrogen bonds (black lines); hydrophobic (purple lines); aromatic (orange lines); aromatic–sulphur (turquoise lines); and cation–pi (blue lines) interactions.

Next, the analysis was applied to the interprotomer interfaces, formed between two adjacent protomer subunits of a single pentamer. Thirty-four residues were identified as hotspots within a single interprotomer interface (Figure 5) by two or more of the prediction programs (Table S2). Thirteen hotspots were residues belonging to VP1, while five, twelve and four residues were predicted as hotspots in the VP2–4 proteins, respectively. Hotspots were found to be more prevalent between VP1 and VP3, followed by VP2 and VP3, and then between two VP1 protein subunits (Figure 5B,C). Protein interaction analysis confirmed that all residues predicted as hotspots form noncovalent contacts with partner residues across the interprotomer interface. The analysis revealed that almost half of the hotspot residues formed interactions with residues that were also predicted as hotspots and that 21 hotspots formed interactions with more than one partner residue across the interface. PIC and jsPISA revealed that hydrophobic interactions are most common between hotspots and partner residues of the VP1–VP1 and VP2–VP3 interfaces, while hydrogen bonds are more common between residues at the VP1–VP3 interface (Figure 5C).

Several hotspots form interactions with multiple residues from different capsid proteins across the interprotomer interface, although not all interactions were predicted as being critical for stability of the interface. For example, residue Y25 in VP4 was classified as a hotspot at the VP4–VP4 interface, but also forms interactions with P19 in VP3 across the interprotomer interface which are not predicted to be critical for stability. Residue Y29 in VP4 is a predicted hotspot in the VP1–VP4 and VP3–VP4 interfaces, but not in the interface between two VP4 proteins. Furthermore, VP1 residue R171 forms hydrogen bonds with residues G101, I224 and G225 across the VP1–VP3 interface where it is considered as a hotspot, but also forms cation–pi interactions with F36 across the VP1–VP1 interface where it is not classified as a stabilizing hotspot residue. Similarly, VP1 residue W176 is a hotspot involved in stabilizing interactions with residues A135, Q184 and R237 across the VP1–VP1 interface but is not a predicted hotspot in the VP1–VP2 interface where contacts are formed with F14 (VP2) (Figure 5C and Table S2). Only two hotspot residues predicted at the interprotomer interfaces of TMEV are known to be functionally important. Notably, both residues are located within a pit involved in binding the TMEV glycoprotein co-receptor (Table 5).

#### 3.2.2. Interprotomer Hotspot Residues in TMEV that are Conserved with Residues in Other Viruses of the Family

**Figure 6:**
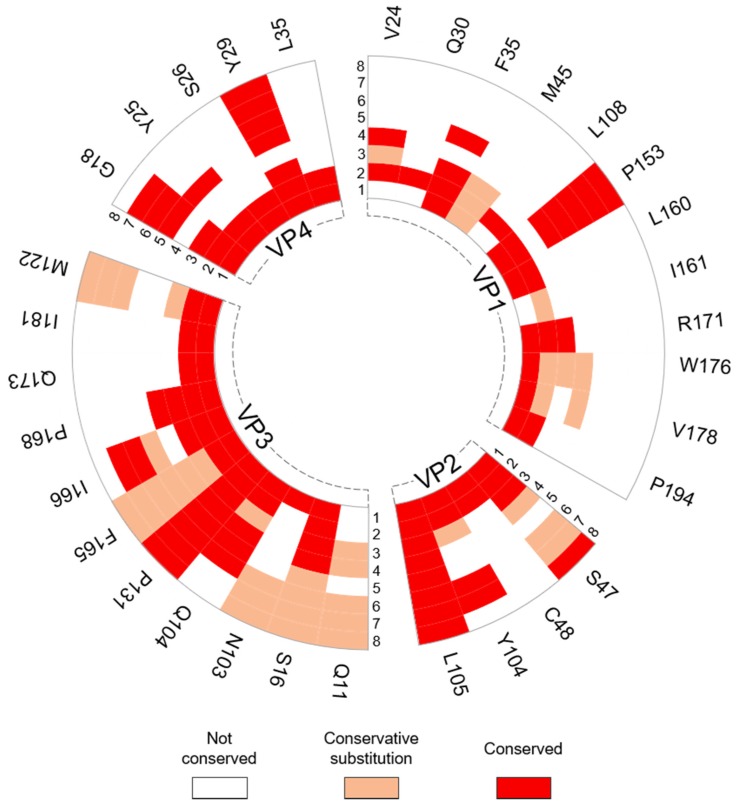
Radial map of the conservation of TMEV interprotomer residues identified as hotspots across respective picornavirus species. The individual TMEV capsid proteins were submitted to ENDScript2 for conservation analysis. The degree of conservation for each TMEV hotspot was compared in eight representative picornaviruses across four genera. Identity of viruses: 1) *Cardiovirus B* (SAFV; PDB: 5CFC/5A8F); 2) *Cardiovirus A* (EMCV; PDB: 2MEV); 3) *Senecavirus* (SVV-1; PDB: 3CJI); 4) *Equine Rhinitis A Virus* (ERAV; PDB: 2WFF); 5) *Foot-and-mouth disease virus* (FMDV; PDB: 5ACA); 6) *Enterovirus A* (EV-71; PDB: 3VBS); 7) *Enterovirus C* (PV-1; PDB: 2PLV); 8) *Rhinovirus A* (HRV-3; PDB: 1RHI). Full alignments are available in Supplementary Figures S1–S4.

Conservation analysis using ENDscript2 revealed that 32 of the 34 hotspot residues in the interprotomer interface are conserved with residues in the capsid proteins of at least one other representative picornavirus (Figure 6). Ten hotspots were only conserved in the *Cardiovirus* genus, while 22 were conserved in viruses from other genera. Hotspot residue Q11 belonging to VP3 was not conserved in encephalomyocarditis virus (EMCV) or Saffold virus (SAFV), but conservative substitutions were present at this position in representative viruses from the other genera. Hotspots S47, L105 (VP2), S16, Q104, P131, F165, I166, (VP3), G18 and Y29 (VP4) were either universally conserved or were subject to conservative mutation in at least one representative from the *Cardio*-, *Seneca*-, *Aphtho*- and *Enterovirus* genera. Several interprotomer hotspot residues were conserved with residues that were previously reported to undergo conformational transitions or to be involved in contacts with the viral genome in other picornaviruses (Table 6).

### 3.3. Analysis of the Residues that Contribute to the Interpentamer Interfaces of TMEV GDVII

#### 3.3.1. Predicted Hotspot Residues at the Interpentamer Interfaces

**Figure 7:**
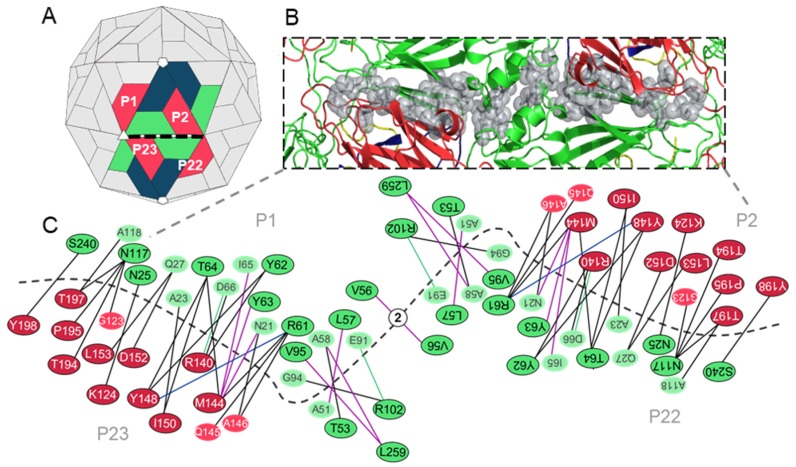
Schematic network of hotspot residues and their interacting partners at the interpentamer interfaces of the TMEV GDVII capsid. (**A**) Structure of the picornavirus capsid showing four protomers from two pentamers that form the interpentamer interface (dashed black line). (**B**) Structural cartoon mapping of predicted hotspot residues (shown as grey spheres) at the interpentamer interfaces. (**C**) Network of hotspot residues and the contacts made with their residue binding partners at the interpentamer interface. Hotspot residues are outlined in black; partner non-hotspots are not outlined. VP1 residues (blue); VP2 residues (green); VP3 residues (red); VP4 residues (yellow). Noncovalent interactions between hotspots and partners are shown as solid lines: hydrogen bonds (black lines); hydrophobic (purple lines); cation–pi (blue lines); and ionic (green lines) interactions.

The interpentamer interfaces are located between the two-fold and three-fold axes of the capsid and involve VP2 subunits of two opposing protomers and VP3 proteins from the adjacent protomers of each pentamer. Thus, the pentamer interfaces are formed by four protomers that can be divided at the two-fold axes into two identical but inverse halves. Twenty-four hotspots were identified at the interpentamer interfaces by two or more prediction methods (Table S3), although it is important to reiterate that the 24 hotspots would be doubled along the full pentamer–pentamer interface, as shown in Figure 7.

Eighteen hotspots were identified between the VP2–VP3 interface, with seven and eleven belonging to the two proteins, respectively. Six hotspots were identified between the two VP2 proteins, and no residues were predicted as hotspots between VP2 and VP4 (Figure 7B,C).

Analysis of residue–residue interactions using the PIC and jsPISA web servers confirmed that all predicted hotspots form noncovalent contacts with residues across the pentameric interface. Furthermore, the analysis indicated that most of the hotspots form hydrogen bonds with partner residues, although some hotspots are involved in hydrophobic interactions nearer to the two-fold axis. Interestingly, all hotspots, excluding T53, L57, R102 (VP2), D152 and L153 (VP3), form interactions with other hotspot residues, and thirteen of the 24 hotspots only form interactions with one partner residue across the interface (Figure 7C).

#### 3.3.2. Interpentamer Hotspot Residues in TMEV that are Conserved with Residues in Other Viruses of the Family

**Figure 8:**
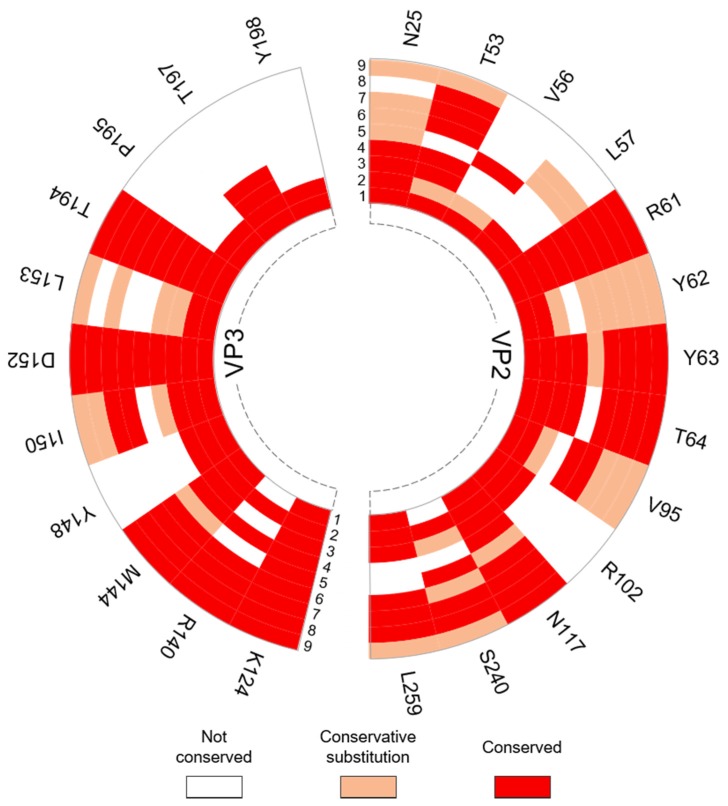
Radial map of the conservation of TMEV interpentamer residues identified as hotspots across respective picornavirus species. The individual TMEV capsid proteins were submitted to ENDScript2 for conservation analysis. The degree of conservation for each TMEV hotspot was compared across nine representative picornaviruses across four genera. Identity of viruses: 1) *Cardiovirus B* (SAFV; PDB: 5CFC/5A8F); 2) *Cardiovirus A* (EMCV; PDB: 2MEV); 3) *Senecavirus* (SVV-1; PDB: 3CJI); 4) *Equine Rhinitis A Virus* (ERAV; PDB: 2WFF); 5) *Foot-and-mouth disease virus* (FMDV; PDB: 5ACA); 6) *Enterovirus A* (EV-71; PDB: 3VBS); 7) *Enterovirus C* (PV-1; PDB: 2PLV); 8) *Enterovirus D* (EV-D68; PDB: 4WM7); 9) *Rhinovirus B* (HRV-B14; PDB: 1R08). Full alignments are available in Supplementary Figures S1–S4.

Sequence and structural alignments in ENDscript2 revealed that all hotspots identified at the interpentamer interfaces are conserved with at least one other virus in the picornavirus family (Figure 8).

Aside from hotspots P195 and Y198 (both VP3), which are conserved in representatives from the *Cardiovirus* genus alone, all hotspot residues are conserved in representative viruses from other genera. Residues R61 (VP2), K124, D152 and T194 (VP3) are universally conserved across the cardio-, seneca-, aphtho- and enteroviruses, while residues N25, T53, Y62, Y63, T64, V95, N117, S240 in VP2 and M144, I150 and L153 in VP3 are conserved in or corresponded to residues with conservative mutations in many of the representatives across the different genera.

Sixteen of the 24 hotspot residues are conserved with residues that were previously reported to be important for virus growth, capsid stability and uncoating in related picornaviruses, of these, hotspots R61, Y63, T64 (in VP2), M144, I150, D152, L153 and T194 (in VP3) were conserved with residues that are described as functionally important in viruses from more than one genera (Table 7).

## 4. Discussion

The residues that critically contribute to the stability and assembly of picornavirus capsids remain poorly investigated. We previously identified a network of conserved interacting motifs within the subunit interfaces of enterovirus capsids and used in silico alanine scanning to elucidate hotspot residues within these preserved regions that contribute to the stability, assembly and uncoating of enteroviruses [36]. In the current study we made use of a different strategy for the prediction of hotspots in the TMEV capsid. Hotspots were identified throughout the entire subunit–subunit interface, rather than in conserved motifs alone, and the conservation of TMEV hotspots was determined thereafter. The rationale for this approach was twofold. Firstly, it allows for the identification of nonconserved hotspots at an individual virus level that have evolved to stabilize the capsid in a manner unique to the virus’s life cycle; secondly, it allows for the extraction of hotspot residues that are conserved in related viruses, which may provide insights into family-wide mechanisms of capsid stability. In this study, we integrated two energy- and three feature-based tools for hotspot prediction, as the use of multiple methods in combination has been shown to improve prediction accuracy [32,33,34,35].

The analysis was first applied to identify hotspot residues within the intraprotomer interfaces of the TMEV capsid. Seventy-two residues were predicted as hotspots that contribute to the binding specificity and stability of these protein–protein interfaces. Most of the hotspots were involved in hydrogen bonds or hydrophobic contacts across the VP1–VP3 and VP2–VP3 interfaces; however, interactions between hotspots and partner residues at the VP1–VP2 interface were predominantly hydrophobic, an expected finding considering that this region is largely buried below the surface.

Several of the intraprotomer hotspot residues predicted in this study have previously been reported to contribute to the stability of the TMEV capsid. Unlike most cardioviruses, TMEV is insensitive to pH-induced dissociation due to extensive interactions which maintain the conformation of surface-exposed loops under acidic conditions [49]. In this study, residues W202, W206, F215 (VP1) and Y135 (VP2) which form hydrophobic interactions between the VP1–VP2 interface were detected as stabilizing hotspots. This finding supports earlier suggestions that these residues form a hydrophobic core that stabilizes the VP1 and VP2 loops [49]. Moreover, the screen detected VP2 residue E146 as a hotspot that interacts with non-hotspot D241 (VP1) through hydrogen bonds that were previously speculated to contribute to stability of the VP1 loop [49]. It has been suggested that stabilizing interactions between capsid subunit loops are also important for TMEV pathogenesis [49,57]. We detected residues R94, W95 and V96 (VP1) as hotspots that form interactions with hotspot residues F176 and M178 in VP2. Interactions between these residues were previously demonstrated to be essential for stability of the VP1-2 loops and the virus life cycle, as substitution of a neighbouring amino acid to a bulkier residue drastically attenuated virus growth and persistence [50,57].

Fifty-seven of the intraprotomer hotspot residues were conserved with at least one other picornavirus analysed, and many corresponded to residues that were reported to function in the capsid stability or the life cycle of these viruses. VP1 hotspots K241 and R249 were conserved in the cardioviruses, enteroviruses and FMDV. Residues K256 and R264 corresponding to K241 and R249 were previously found to be vital for EV-71 replication [20], while hotspots Y124, N204, F246 and R249 (VP1) are conserved with energetically important residues that were predicted to contribute to the stability of intraprotomer interfaces in the enteroviruses [36]. Several studies have recently speculated that interactions between VP4, the N-termini of VP1-3 and viral RNA also influence the dynamics of capsid stability [51,58,59,60,61]. In this study, protein–RNA interactions were not analysed; however, it is interesting to note that hotspot K241 (VP1) corresponds to residue R202 in human parechovirus 3 (HPeV-3) that was previously reported to interact with the viral genome [51].

The pentamer subunits are highly stable intermediates during capsid assembly and uncoating [10,62]. It is speculated that evolution has favoured strong interactions between the interprotomer interfaces, as pentamers are not required to dissociate into protomers during uncoating, and strong interactions promote higher concentrations of intact pentamers for efficient capsid assembly [9]. To identify hotspot residues that contribute to the stability of the interprotomer interfaces, a complex of two adjacent protomers was submitted for analysis. Thirty-four residues were predicted as hotspots, which mainly form hydrogen bonds and hydrophobic interactions with partner residues across the interface. Hotspot residues P153 (VP1) and I181 (VP3) which interact with partner residues in VP3 and VP2 respectively, were previously shown to be located within the TMEV receptor binding site [52]. This is unsurprising considering that the receptor binding sites of most picornaviruses, such as the canyons of enteroviruses, involve residues that traverse the protomer–protomer interfaces [63,64]. Furthermore, P153 was previously reported to be directly involved in binding the TMEV co-receptor, following a series of in vitro substitution experiments that attenuated or abolished receptor binding and infectivity [52]. The uncoating mechanism of TMEV is poorly understood, and the insensitivity of TMEV to acidic conditions suggests that uncoating may be induced by receptor binding, unlike other cardioviruses [49,65]. It is tempting to speculate that the binding of the TMEV co-receptor, or unknown primary receptor, to energetically important hotspots including P153 may destabilize the interface and contribute to the induction of uncoating. Conversely, these hotspots may maintain the binding site in the correct conformation for receptor interactions but may not be destabilized upon binding.

It is unclear as to whether all cardioviruses form expanded intermediates during uncoating, as A-particles have only been observed for SAFV-3 [53]. Interestingly, several interprotomer hotspot residues belonging to VP1 and 2 correspond to residues in the SAFV-3 A-particle that maintain their interactions during expansion where they surround the edges of the five-fold pore. In TMEV these hotspots are involved in strong hydrophobic interactions with their partner residues. A second set of TMEV hotspots in VP1 and 3 that form weaker hydrogen bonds with their partner residues correspond to residues in SAFV-3 that undergo conformational changes to yield the pore [53]. It is tempting to speculate that these conserved residues function similarly in TMEV. Residues at the interprotomer interfaces in picornaviruses are variable and contribute to a variety of topological features that are species-specific [49,64,66]. Despite the variability of amino acids at the interprotomer interfaces, 22 of the 34 residues predicted as hotspots were conserved in representative viruses from other genera, suggesting that the mechanisms that regulate the stability of the interprotomer interfaces may be somewhat conserved across the picornavirus family.

It is generally accepted that the interfaces between pentamer subunits are major sites of structural rearrangement during capsid uncoating. For example, studies have widely documented the formation of pores at these interfaces [67,68,69,70,71,72,73] or the loss of one or more pentamer subunit(s) [53,58,74,75] upon exposure of the picornavirus capsid to denaturing conditions. To identify the hotspot residues that are critical to the binding specificity and stability of the pentamer interfaces, complexes of two opposing protomers at the two-fold axis and two adjacent protomers at the three-fold axis were analysed. Twenty-four residues were identified as stabilizing hotspots at these interfaces; however, the residues are doubled across the entire pentamer interface as four protomers are involved. Most of the hotspot residues formed hydrogen bonds with partner residues across the interface, although hydrophobic interactions were more common near to the two-fold axis, consistent with suggestions that weak noncovalent interactions mediate pentamer–pentamer binding [9,76,77]. Unlike the intraprotomer and interprotomer interfaces, all hotspot residues at the interpentamer interface were conserved in at least one other virus of the family. Residues P195 and Y198 in VP3 were the only hotspots conserved in cardiovirus representatives alone, while hotspots R61 (VP2), K124, D152 and T194 (VP3) were universally conserved in all representatives from the cardio-, seneca-, aphtho- and enteroviruses. Interestingly, these residues correspond to residues R60, R120, D148 and T190 in FMDV, respectively, which were shown to be fundamental for virus growth and capsid stability following the in vitro substitution of these residues to alanine [11]. TMEV hotspot Y62 (VP2) is replaced in the seneca-, aphtho- and enteroviruses by residues with similar properties. It was previously shown that substitution of F62 in FMDV Type O and SAT 2 to the tyrosine observed in TMEV improved the thermostability of FMDV [55].

Most of the hotspots identified at the interpentamer interfaces in this study were conserved with residues in other picornaviruses that were previously predicted to stabilize the capsid. For example, hotspots N25, R61, Y62, Y63, T64, V95, R102, N117, S240 (VP2), M144, Y148, I150, D152, L153, T194 and T197 (VP3) correspond to residues in Seneca Valley virus 1 (SVV-1) that were proposed to form stabilizing interactions across the pentamer–pentamer interface [54], while hotspot residues R61, T64, (VP2), M144 and D152 (VP3) were conserved with residues that were predicted to be important for the stability of the pentamer interfaces of enteroviruses [36]. Furthermore, hotspots M144, I150, D152 and L153 (VP3) are conserved with residues in human rhinovirus (HRV)-2 that become disordered during uncoating and RNA release [56]. HRVs are prone to dissociation in acidic conditions, and histidine residues at the two-fold axis have been implicated in this sensitivity. Residues in HRV-B14 corresponding to TMEV hotspots R61 (VP2) and K124 (VP3) form interactions with histidine residues and become destabilized at mild acidity [78]. However, in TMEV, these hotspots form interactions with partner residues which resist protonation under mildly acidic conditions. Furthermore, hotspot R61 forms cation–pi interactions with hotspot Y148 (VP3) in TMEV but in HRV-B14 Y148 is replaced with the acid-sensitive H150. These findings may partially explain the different pH stabilities of these viruses.

## 5. Conclusions

This study presents a comprehensive analysis of the hotspot residues that contribute to the stability of the intraprotomer, interprotomer and interpentamer interfaces of the TMEV capsid. Many of the predicted hotspots, particularly those between pentamers, were conserved with residues reported to be important for virus growth and capsid stability in representative viruses across the family. This finding suggests that the molecular determinants of capsid stability may be conserved across the picornaviruses, providing a logical basis for further investigations into the significance of these residues to aspects of the virus life cycle. For example, our preliminary in vitro mutagenesis experiments have demonstrated that conserved hotspot residues predicted in the interpentamer interface are required for the development of cytopathic effect in permissive cells, but not for viral replication or protein synthesis. Finally, the analysis presented here can be applied to any icosahedral virus as a first step in identifying residues that significantly contribute to the stability and assembly of the viral capsid, which represent potential antiviral targets.

## Figures and Tables

**Table 1:** The web servers and databases used for the prediction of hotspot residues in this study.

Web Server/Database	Strategy	Technique	Ref.
ROBETTA http://www.robetta.org/alascansubmit.jsp	Energy-based:	Computational alanine scanning: Interfacial residues are individually substituted to alanine. The Δ binding free energy of the complex and Δ protein stability are calculated. Hotspots are defined as residues that increase the binding free energy ≥1 kcal/mol following substitution to alanine.	[23]
	Free energy function.		
PPCheck http://caps.ncbs.res.in/ppcheck/	Energy- and feature-based:	Energy scoring scheme: Calculates and imparts pseudoenergies to noncovalent interactions in protein–protein interfaces. These energies are weighted with features to identify hotspot residues.	[40]
	Energy scoring, extent of spatial residue interaction, extent of energy contribution.		
PredHS http://predhs.denglab.org/	Feature- and energy-based:	Machine Learning: Euclidian and Voronoi neighbourhoods are used to weight features and generate individual residue scores. Hotspots are defined as residues with scores >0.	[41]
	38 sequence-, structure- and energy-based features.		
KFC2 https://mitchell-web.ornl.gov/KFC_Server/upload.php	Feature-based:	Machine Learning: Various features are assessed by two models built using support vector machines. Residues which are detected as hotspots are highlighted.	[42,43]
	Residue size, packing density, solvent accessibility, hydrophobicity, flexibility.		
HotRegion/Hotpoint http://prism.ccbb.ku.edu.tr/hotregion	Feature-based:	Machine Learning: HotPoint assesses features using an empirical model and defines a residue as a hotspot if ASA values are ≤20% and contact potential values ≥18.0. HotRegion creates a network of these hotspots and highlights those which form contacts.	[44]
	Solvent accessibility (ASA) and residue contact potential/known residue pair energies.		

**Table 2:** Web servers used for the prediction of interactions across the subunit interfaces in this study.

Web Server	Technique	Interactions and Cutoff Distances	Ref.
jsPISA http://www.ccp4.ac.uk/pisa	Uses seven parameters to identify interfaces. Solvation energy, binding energy, hydrophobic *p*-value, number and type of contacts are then determined.	hydrogen bonds; salt bridges; disulphide bonds	[46]
PIC http://pic.mbu.iisc.ernet.in/	Predicts residue–residue interactions at protein–protein interfaces from the atomic coordinates of the input complex using standard widely accepted criteria.	hydrogen bonds; hydrophobic interactions (5 Å); cation-pi and ionic interactions (6 Å); disulphide bonds and aromatic–aromatic interactions bonds (4.5–7 Å); aromatic–sulphur interactions (5.3 Å)	[45]

**Table 3:** Intraprotomer hotspot residues in TMEV with previously known functions.

Hotspot Residue	Known Function	Ref.
VP1: R94, W95 & V96 VP2: F176 & M178	Reside on VP1 loop II and VP2 Puff A. Interactions between these residues are predicted to increase the stability of these loops. The substitution of A101 within the VP1 loop to tryptophan disrupted interactions between these residues and reduced virus yield and persistence.	[49,50]
VP1: W202, W206 & F215 VP2: Y135 & E146	W202-Y135 form a strong hydrophobic core which stabilizes the TMEV VP1 foot-and-mouth-disease virus (FMDV) loop. E146 on VP2 puff B forms hydrophobic interactions with a non-hotspot on the FMDV loop of VP1. These interactions allow TMEV, unlike other cardioviruses, to remain stable under a broad range of pH conditions.	[49]
VP1: F254 VP3: R100	Reside in receptor binding site and were predicted to be involved in binding to the TMEV co-receptor heparan sulphate.	[38]
VP1: V245, F246, R249, L252, F253, F254, W256, T260 & I272	Form part of the VP1 C-terminal loop located over the receptor binding site.	[38]

**Table 4:** Intraprotomer hotspot residues in TMEV conserved with residues in related viruses with previously identified roles.

Hotspot Residue	Corresponding Residues in Related Viruses with Known Function(s)	Ref.
VP1: K241 & R249	Correspond to residues K256 and R264 in enterovirus 71 (EV-71), which have been shown to be necessary for virus replication. In vitro substitution of either residue to alanine was lethal as virus could not be recovered.	[20]
VP1: Y124, N204, F246 & R249	Conserved with energetically important residues Y128, D206, W261, and R264 (VP1) that form part of the intraprotomer interfaces of EV-71 which were found in a conserved motif within the enteroviruses.	[36]
VP1: K241	Corresponds to residue R202 in the VP1 protein of human parechovirus 3 (HPeV-3) that is known to be involved in interactions with the viral genome.	[51]

**Table 5:** Interprotomer hotspot residues in TMEV with previously known functions.

Hotspot Residue	Known Function	Ref.
VP1: P153 VP3: I181	These residues are exposed at the bottom of the putative receptor binding site in TMEV. P153 was previously shown to be critical for binding the unknown glycoprotein receptor.	[52]

**Table 6:** Interprotomer hotspot residues in TMEV conserved with residues in related viruses with previously identified roles.

Hotspot Residue	Corresponding Residues in Related Viruses with Known Role(s)	Ref.
VP1: P153, VP3: N103, Q104, Q173, I181 & M222	Conserved with residues in Saffold virus 3 (SAFV-3) that undergo conformational transitions to form a pore at the protomer–protomer interface in the expanded particle, which is thought to be involved in RNA release.	[53]
VP3: S16	Corresponds to residue T47 in the VP3 protein of HPeV-3 that is known to make contacts with the RNA genome.	[51]

**Table 7:** Interpentamer hotspot residues in TMEV conserved with residues in related viruses with previously identified roles.

Hotspot Residue	Corresponding Residues in Related Viruses with Known Function(s)	Ref.
VP2: N25, R61, Y62 (W in SVV-1), Y63, T64, V95 (A in SVV-1), R102, N117, S240 (T in SVV-1) VP3: M144, Y148, I150, D152, L153 (I in SVV-1), T194, T197	Conserved with residues at the pentamer interfaces of the Seneca Valley virus 1 (SVV-1) mature capsid that are predicted to form interactions across the interface.	[54]
VP2: R61 VP3: K124, D152 & T194	Correspond to residues R60 (VP2), R120, D148 and T190 (VP3) in FMDV, respectively, that were found to be important for virus growth. The in vitro substitution R60A was lethal as the virus could not be recovered. Residue substitutions R120A and D148A attenuated viral growth, yield and plaque size. The substitution of T194A could only be recovered after genotypic reversion.	[11]
VP2: Y63	Corresponds to F62 in FMDV SAT2. Mutation of F62 to tyrosine, as seen in TMEV, increased the stability of the FMDV particle.	[55]
VP2: R61 & T64 VP3: M144 & D152	Conserved with energetically important residues within conserved interacting motifs at the two-fold axes of enteroviruses.	[36]
VP3: M144, I150, D152 & L153	Conserved with residues in human rhinovirus (HRV)-2 VP3 at the pentamer interface that become disordered during capsid uncoating and RNA release.	[56]

## Supplementary Materials

The following are available online at https://www.mdpi.com/1999-4915/12/4/387/s1, Table S1: Programs that detected hotspot residues at the intraprotomer interface. Table S2: Programs that detected hotspot residues at the interprotomer interface. Table S3: Programs that detected hotspot residues at the interpentamer interface. Figure S1. Sequence-based alignment of TMEV, cardiovirus, senecavirus, aphthovirus and enterovirus VP1 proteins. Figure S2. Sequence- and structure-based alignment of TMEV VP2 with cardiovirus, senecavirus, aphthovirus and enterovirus representatives. Figure S3: Sequence- and structure-based alignment of TMEV VP3 with cardiovirus, senecavirus, aphthovirus and enterovirus representatives. Figure S4: Sequence-based alignment of TMEV, aphthovirus, cardiovirus and enterovirus VP4 proteins.
