# Supplementary material for: The In Silico Prediction of Hotspot Residues that Contribute to the Structural Stability of Subunit Interfaces of a Picornavirus Capsid
Source: Viruses. 2020 Mar 31;12(4):387. doi: 10.3390/v12040387 (PMC7232237; doi:10.3390/v12040387)
Supplement: Supplementary file 1 [file viruses-12-00387-s001.pdf]

Table S1. Programs that detected hotspot residues at the intraprotomer interface [1].

Protein	Residue	KFC2	ROBETTA	Hot Region	PredHS	PPCheck
VP1-VP2 Interface						
VP1	E6				■	■
	R94	■	■	■	■	
	W95	■	■	■	■	■
	V96	■	■	■		
	Y124	■	■	■	■	
	W202	■	■		■	■
	F203	■	■	■		
	N204	■	■	■	■	■
	W206	■	■	■	■	
	F215	■	■	■	■	■
	R249	■	■		■	■
	L252	■	■		■	
	F253	■	■		■	
	T260		■		■	
VP2	E133	■	■	■	■	
	F134		■	■		
	Y135	■	■	■		
	T136		■	■		
	E146		■		■	
	R168	■	■		■	
	Y169	■	■			
	F175	■	■	■	■	■
	F176	■	■	■	■	■
	M178	■		■	■	■
	Q185		■		■	
	Y189	■	■		■	
	V211		■		■	
VP1-VP3 Interface						
VP1	T31		■		■	■
	V33	■	■	■	■	
	F36	■	■	■		
	Y114		■	■		
	C118		■	■		
	F119	■	■	■	■	■
	F122	■	■	■	■	■
	F187	■	■	■		
	V189		■	■		
	R239	■	■			
	V245	■	■			

	F246	■	■	■		
	F254	■	■		■	
	W256	■	■	■	■	■
	I272		■		■	■
VP3	I25		■		■	■
	F41	■	■	■	■	■
	L44	■	■	■	■	■
	L47		■	■		
	V98	■	■	■		
	R100		■	■		
	F102	■	■	■	■	
	R106	■	■			
	L112	■	■	■		
	V114		■	■		
	F159	■	■	■		
	N160	■	■			
	F161	■		■	■	
	D217		■		■	
	W229	■	■		■	
	Q232		■		■	
VP1-VP4 Interface						
VP1	K9				■	■
	D128		■		■	
	K241		■		■	
VP2-VP3 Interface						
VP2	W184	■		■	■	
	Q185		■		■	
	R197	■	■		■	
	L230	■	■	■		
	S231		■	■	■	
VP3	F53	■		■	■	
	F115	■	■	■	■	
	T116		■	■	■	
	L209	■	■	■		
VP2-VP4 Interface						
VP2	R32		■		■	
	L33		■		■	
	Y36		■		■	
VP3-VP4 Interface						
No residues predicted to be important for stability of interface						

Table S1. Footnotes:

Grey blocks indicate where a program has predicted a residue to be a hotspot. Residues that were detected as hotspots by two or more programs are shown.

Table S2. Programs that detected hotspot residues at the interprotomer interface [1].

Protein	Residue	KFC2	ROBETTA	Hot Region	PredHS	PPCheck
VP1-VP1						
VP1	L160		■	■		■
	I161		■	■		■
	Y163		■		■	
	W176	■	■		■	■
	V178		■			■
	M45	■	■			
	L108		■	■		
VP1-VP2						
VP1	V24				■	■
VP1-VP3						
VP1	Q30			■		■
	F35				■	■
	P153				■	■
	R171		■			■
	P194				■	■
VP3	Q11		■		■	
	C13		■		■	
	S16		■		■	
	N103	■	■	■	■	
	Q104	■		■	■	
	Q173		■	■	■	
	I181		■			■
	M222	■	■	■	■	■
VP1-VP4						
VP4	Y29				■	■
	L35		■		■	■
VP2-VP3						
VP2	S47			■	■	■
	C48		■	■		
	Y104	■		■	■	
	L105	■	■	■	■	
VP3	P131				■	■
	F165	■	■	■	■	
	I166	■	■	■	■	■
	P168				■	■
VP3-VP3						
No residues predicted to be important for stability of interface						
VP3-VP4						
VP4	Y25				■	■

	Y29					
VP4-VP4						
VP4	G18					
	S26					

Table S2. Footnotes:

Grey blocks indicate where a program has predicted a residue to be a hotspot. Residues that were detected as hotspots by two or more programs are shown.

Table S3. Programs that detected hotspot residues at the interpentamer interface [1].

Protein	Residue	KFC2	ROBETTA	Hot Region	PredHS	PPCheck
VP1-VP2						
No residues predicted to be important for stability of interface						
VP2-VP2						
VP2	T53		■	■	■	■
	V56		■	■	■	■
	L57			■	■	■
	V95	■			■	■
	R102	■	■		■	■
	L259		■	■	■	■
VP2-VP3						
VP2	N25				■	■
	R61	■	■	■	■	
	Y62			■	■	
	Y63	■	■	■		
	T64	■	■	■	■	
	N117		■		■	■
	S240	■			■	
VP3	K124	■		■	■	■
	R140		■	■		
	M144	■		■	■	
	Y148	■	■	■	■	■
	I150	■	■	■	■	■
	D152	■		■	■	
	L153		■	■		
	T194	■	■		■	■
	P195	■			■	
	T197	■	■	■	■	
	Y198	■	■	■	■	■
VP2-4						
No residues predicted to be important for stability of interface						

Table S3. Footnotes:

Grey blocks indicate where a program has predicted a residue to be a hotspot. Residues that were detected as hotspots by two or more programs are shown.

```
                                                                                                1         10        20
GDVII_A                            .............................................................GIDNAEKGKVSNDDASVDFVAEPVKLPEN
2MEV_1|PDBID|CHAIN|SEQUENCE        .............................................................GVENAEKGVTENTDATADFVAQPVYLPEN
5A8F_A|PDBID|CHAIN|SEQUENCE        ..........................................................................................
3CJI_A|PDBID|CHAIN|SEQUENCE        .............................................................STDNAETGVIEAGNTDTDFSGELAAPGSN
1FMD_1|PDBID|CHAIN|SEQUENCE        ...........................................................TT...TTGESADPVTTTVENYGGETQVQRRH
5ACA_1|PDBID|CHAIN|SEQUENCE        ...........................................................TT...SSGEGADVVTTDPSTHGGAVTEKKRV
2XBO_1|PDBID|CHAIN|SEQUENCE        ................................................................VTNVGEDGEPGETEPRHALSPVDMHV
3VBF_A|PDBID|CHAIN|SEQUENCE        GDRVADVIESSIGDSVSR............ALTH..ALPAPTGQNTQVSSHRLDTGKVPALQAAEIGASSNASDESMIETRCVLNSHSTA
1R08_1|PDBID|CHAIN|SEQUENCE        ...........................GLGDELEEVIVEKTKQTVASISSGPKHTQKVPILTANETGATMPVLPSDSIETRTTYMHFNGS
1FPN_1|PDBID|CHAIN|SEQUENCE        ...........................NPVENYIDEVLNEVLVVPNINSSNPTTSNSAPALDAAETGHTSSVQPEDVIETRYVQTSQTRD
1RHI_1|PDBID|CHAIN|SEQUENCE        ...........................GLSDELEEVIVEKTKQTLASVSSGPKHTQSVPALTANETGATLPTRPSDNVETRTTYMHFNGS
2PLV_1|PDBID|CHAIN|SEQUENCE        .................GLGQMLESMIDNTVSSTVGAATSRDALPNTEASGPTHSKEIPALTAVETGATNPLVPSDTVQTRHVVQHRSRS
1D4M_1|PDBID|CHAIN|SEQUENCE        ................GD............VEEA..IERAVVHVADTMRSGPSNSASVPALTAVETGHTSQVTPSDTMQTRHVKNYHSRS
4WM7_A|PDBID|CHAIN|SEQUENCE        .................................V..ESIIKTATDTVKSEINAELGVVPSLNAVETGATSNTEPEEAIQTRTVINQHGVS
consensus>70                       ................................................................e.............e...........

                                   30        40        50        60        70        80        90        100
GDVII_A                            QTRVAFFYDRAVPIGMLRPGQNME.....T.TFSYQE..NDFRLNCLLLTPLPSYCPDSSSGPVRTKAPVQWRWVRSGG..ANGANFPLM
2MEV_1|PDBID|CHAIN|SEQUENCE        QTKVAFFYDRSSPIGAFAVKSGSL.....ESGFAPFS..NKACPNSVILTPGPQFD.....PAYDQLRPQRLTEIWGNGNEETSEVFPLK
5A8F_A|PDBID|CHAIN|SEQUENCE        ....SFFYDRSTLSSVLQSTSDVS.....S.KFTPST..AKNLQNSILLTPLPSDIVN......NSVLPEQERWISFAS..PTTQKPPYK
3CJI_A|PDBID|CHAIN|SEQUENCE        HTNVKFLFDRSRLLNVIKVLEKDAVFPRPF.PTQEGA..QQDDGYFCLLTPRPTVAS............RPATRFGLYA.NPSGSGVLAN
1FMD_1|PDBID|CHAIN|SEQUENCE        HTDVAFVLDRFVKVTVS...........................DNQHTLDVMQA...................................
5ACA_1|PDBID|CHAIN|SEQUENCE        HTDVAFVMDRFTHV.LTN..........................RTAFAVDLMDT...................................
2XBO_1|PDBID|CHAIN|SEQUENCE        HTDVSFLLDRFFDVETLELSN...................LTGSPATHVLDPFGS...................................
3VBF_A|PDBID|CHAIN|SEQUENCE        ETTLDSFFSRAGLVGEIDLPL............KGTT..NPNGYANWDIDIT......................................
1R08_1|PDBID|CHAIN|SEQUENCE        ETDVECFLGRAACVHVTEIQNKDA.....T.GIDNHR..EAKLFNDWKINLS......................................
1FPN_1|PDBID|CHAIN|SEQUENCE        EMSLESFLGRSGCIHESKLEVTL...........ANY..NKENFTVWAINLQ......................................
1RHI_1|PDBID|CHAIN|SEQUENCE        ETDVESFLGRAACVHVTEIKNKNA.....A.GLDNHR..KEGLFNDWKINLS......................................
2PLV_1|PDBID|CHAIN|SEQUENCE        ESSIESFFARGACVTIMTVDNPAS.......T...TN..KDKLFAVWKITYK......................................
1D4M_1|PDBID|CHAIN|SEQUENCE        ESTVENFLGRSACVYMEEYKTTD............ND..VNKKFVAWPINTK......................................
4WM7_A|PDBID|CHAIN|SEQUENCE        ETLVENFLGRAALVSKKSFEYKNH.....A.S...SSAGTHKNFFKWTINTK......................................
consensus>70                       .t.v..f..R...v............................................................................

                                   110       120       130                 140       150       160       170
GDVII_A                            TKQDYAFLCFSPFTYYKCDLEVTVSAMGA............GTVS..SVLRWAPTGAPADVTDQLI........GYTPSLGETRNPHMWI
2MEV_1|PDBID|CHAIN|SEQUENCE        TKQDYSFCLFSPFVYYKCDLEVTLSPHTS............GAHG..LLVRWCPTGTPTKPTTQVL........HEVSSLSEGRTPQVYS
5A8F_A|PDBID|CHAIN|SEQUENCE        TKQDWNFIMFSPFTYYKCDLEVTLSKNDR............ETIS..SVVRYVPCGAPSDLSDQTM........PQTPSLADTRDPHMWV
3CJI_A|PDBID|CHAIN|SEQUENCE        TSLDFNFYSLACFTYFRSDLEVTVVSLEP.............DLE..FAVGWFPSGSEYQASSFVY......DQLHVPFHFTGRTPRAFA
1FMD_1|PDBID|CHAIN|SEQUENCE        HKDNIVGALLRAATYYFSDLEIAVTHT....................GKLTWVPNGAPVSA..................LNNTTNPTAYH
5ACA_1|PDBID|CHAIN|SEQUENCE        NEKTLVGALLRAATYYFCDLEIACLGE.................HERVW..WQPNGAPRTT...................TLRDNPMVFS
2XBO_1|PDBID|CHAIN|SEQUENCE        TAQLAWARLLNTCTYFFSDLELSIQFKFTTT.P.....SSVGEGF..VWVKWFPVGAPTKTTDAWQLEGGGNSVRIQQLAVAGMSPTVVF
3VBF_A|PDBID|CHAIN|SEQUENCE        .GYAQMRRKVELFTYMRFDAEFTFVACTP........TGEVVPQL..LQYMFVPPGAPKPDSR.............ESLAWQTATNPSVFV
1R08_1|PDBID|CHAIN|SEQUENCE        .SLVQLRRKLELFTYVRFDSEYTILATASQP...DS.ANYSSNLV..VQAMYVPPGAPNPKEW............DDYTWQSASNPSVFF
1FPN_1|PDBID|CHAIN|SEQUENCE        .EMAQIRRKFELFTYTRFDSEITLVPCISAL...S...QDIGHIT..MQYMYVPPGAPVPNSR............DDYAWQSGTNASVFW
1RHI_1|PDBID|CHAIN|SEQUENCE        .SLVQLRRKLELFTYVRFDSEYTILATASQP...EA.SSYSSNLT..VQAMYVPPGAPNPKEW............DDYTWQSASNPSVFF
2PLV_1|PDBID|CHAIN|SEQUENCE        .DTVQLRRKLEFFTYSRFDMELTFVVTANFT.E..TNNGHALNQV..YQIMYVPPGAPVPEKW............DDYTWQTSSNPSIFY
1D4M_1|PDBID|CHAIN|SEQUENCE        .QMVQMRRKLEMFTYLRFDMEVTFVITSRQD.PGTTLAQDMPVLT..HQIMYVPPGGPIPAKV............DDYAWQTSTNPSIFW
4WM7_A|PDBID|CHAIN|SEQUENCE        .SFVQLRRKLELFTYLRFDAEITILTTVAVNGNNDSTYMGLPDLT..LQAMFVPTGALTPKEQ............DSFHWQSGSNASVFF
consensus>70                       ............ftY...D.E.t..............................P.Gap..........................np.vf.
```

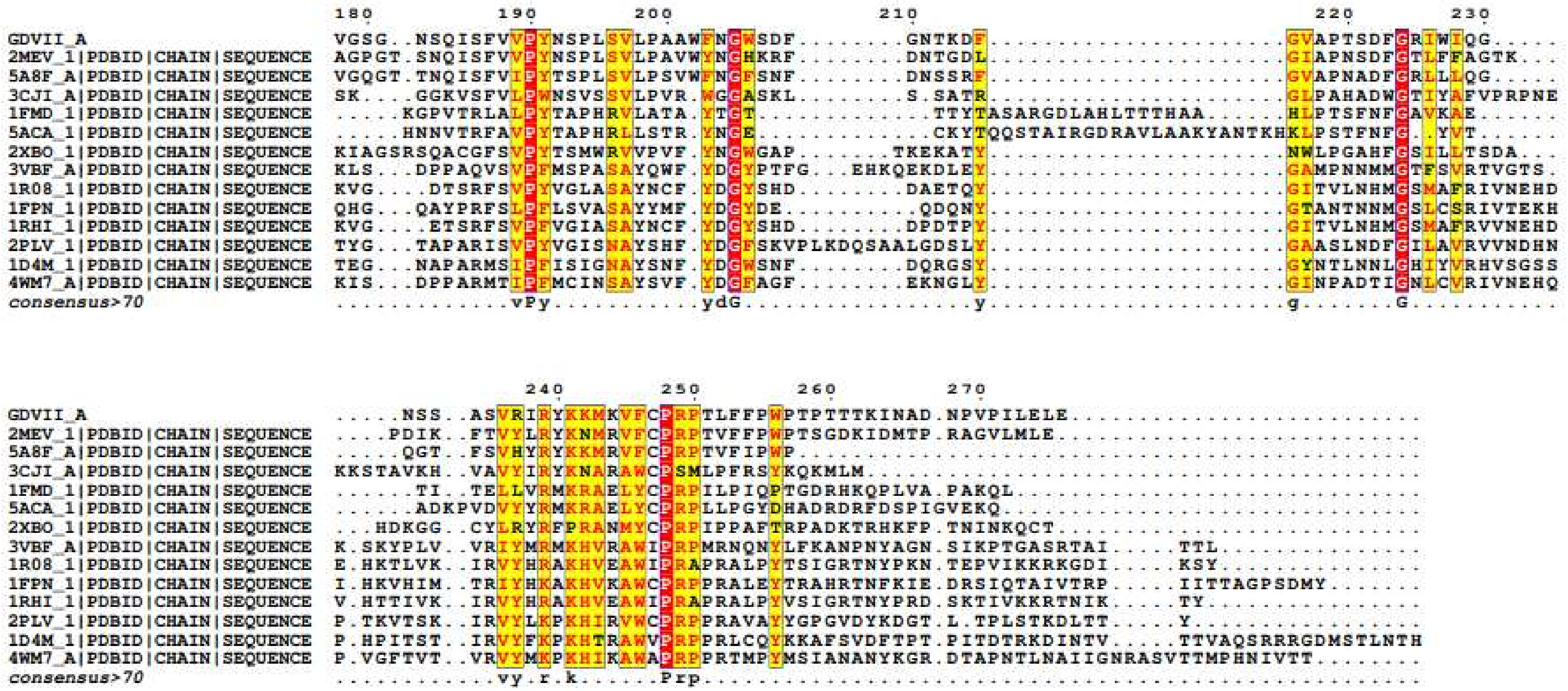

Figure S1. Sequence-based alignment of TMEV, cardiovirus, senecavirus, aphthovirus and enterovirus VP1 proteins [1]. VP1 protein sequences were obtained from the PDB database: cardioviruses MEV and SAFV (PDB: 2MEV, 5A8F); senecavirus SVV-1 (PDB: 3CJI); aphthoviruses FMDV type C and SAT 2, ERAV (PDB: 1FMD, 5ACA, 2WFF); and enteroviruses EV-71, HRV-B14, HRV-2, HRV-3, PV-1, CV-A9, EV-D68 (PDB: 3VBF, 1R08, 1FPN, 1RHI, 2PLV, 1D4M, 4WM7); and were submitted to ESpript (built into ENDscript) to produce a multiple sequence alignment of the amino acid sequences.

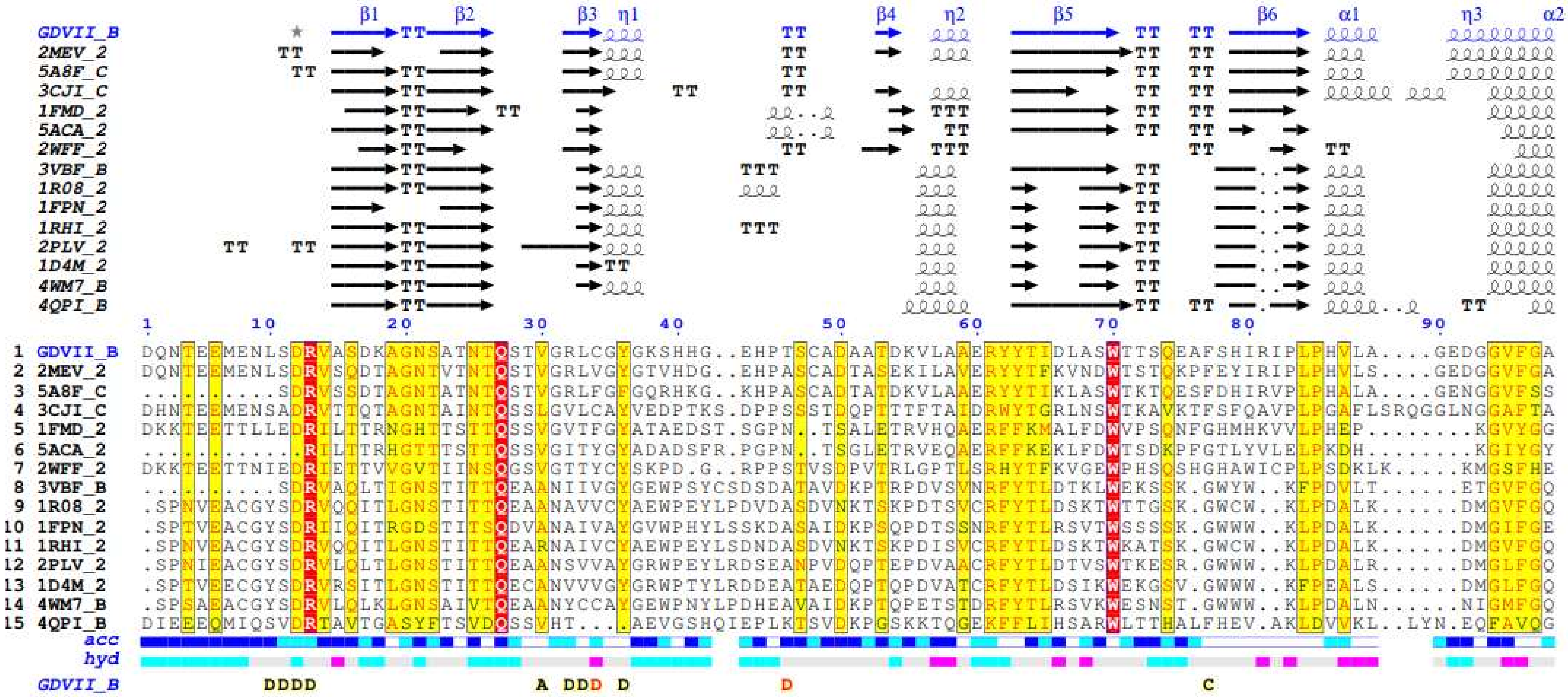
β1 β2 β3 η1 β4 η2 β5 β6 α1 η3 α2
GDVII_B
2MEV_2
5A8F_C
3CJI_C
1FMD_2
5ACA_2
2WFF_2
3VBF_B
1R08_2
1FPN_2
1RHI_2
2PLV_2
1D4M_2
4WM7_B
4QPI_B
1 10 20 30 40 50 60 70 80 90
1 GDVII_B DQNTEEMENLSDRVASDKAGNSATNTQSTVGRLCGYGKSHHG..EHPTSCADAATDKVLAAERYYTIDLASWTTSQEAFSHIRIPLPHVLA....GEDGGVFGA
2 2MEV_2 DQNTEEMENLSDRVSQDTAGNTVTNTQSTVGRLVGYGTVHDG..EHPASCADTASEKILAVERYYTFKVNDWTSTQKPFEYIRIPLPHVLS....GEDGGVFGA
3 5A8F_C ..........SDRVSSDTAGNTATNTQSTVGRLFGFGQRHKG..KHPASCADTATDKVLAAERYYTIKLASWTKTQESFDHIRVPLPHALA....GENGGVFSS
4 3CJI_C DHNTEEMENSADRVTTQTAGNTAINTQSSLGVLCAYVEDPTKS.DPPSSSTDQPTTTFTAIDRWYTGRLNSWTKAVKTFSFQAVPLPGAFLSRQGGLNGGAFTA
5 1FMD_2 DKKTEETTLLEDRILTTRNGHTTSTTQSSVGVTFGYATAEDST.SGPN..TSALETRVHQAERFFKMALFDWVPSQNFGHMHKVVLPHEP.........KGVYGG
6 5ACA_2 ...........RILTTRHGTTTSTTQSSVGITYGYADADSFR.PGPN..TSGLETRVEQAERFFKEKLFDWTSDKPFGTLYVLELPKDH.........KGIYGY
7 2WFF_2 DKKTEETTNIEDRIETTVVGVTIINSQGSVGTTYCYSKPD.G..RPPSTVSDPVTRLGPTLSRHYTFKVGEWPHSQSHGHAWICPLPSDKLK.....KMGSFHE
8 3VBF_B .........SDRVAQLTIGNSTITTQEAANIIVGYGEWPSYCSDSDATAVDKPTRPDVSVNRFYTLDTKLWEKSSK.GWYW..KFPDVLT......ETGVFGQ
9 1R08_2 .SPNVEACGYSDRVQQITLGNSTITTQEAANAVVCYAEWPEYLPDVDASDVNKTSKPDTSVCRFYTLDSKTWTTGSK.GWCW..KLPDALK......DMGVFGQ
10 1FPN_2 .SPTVEACGYSDRIIQITRGDSTITSQDVANAIVAYGVWPHYLSSKDASAIDKPSQPDTSSNRFYTLRSVTWSSSSK.GWWW..KLPDALK......DMGIFGE
11 1RHI_2 .SPNVEACGYSDRVQQITLGNSTITTQEARNAIVCYAEWPEYLSDNDASDVNKTSKPDISVCRFYTLDSKTWKATSK.GWCW..KLPDALK......DMGVFGQ
12 2PLV_2 .SPNIEACGYSDRVLQLTLGNSTITTQEAANSVVAYGRWPEYLRDSEANPVDQPTEPDVAACRFYTLDTVSWTKESR.GWWW..KLPDALR......DMGLFGQ
13 1D4M_2 .SPTVEECGYSDRVRSITLGNSTITTQECANVVVGYGRWPTYLRDDEATAEDQPTQPDVATCRFYTLDSIKWEKGSV.GWWW..KFPEALS......DMGLFGQ
14 4WM7_B .SPSAEACGYSDRVLQLKLGNSAIVTQEAANYCCAYGEWPNYLPDHEAVAIDKPTQPETSTDRFYTLRSVKWESNST.GWWW..KLPDALN......NIGMFGQ
15 4QPI_B DIEEEQMIQSVDRTAVTGASYFTSVDQSSVHT....AEVGSHQIEPLKTSVDKPGSKKTQGEKFFLIHSARWLTTHALFHEV.AKLDVVKL..LYN.EQFAVQG
acc
hyd
GDVII_B DDDD A DDD D D C

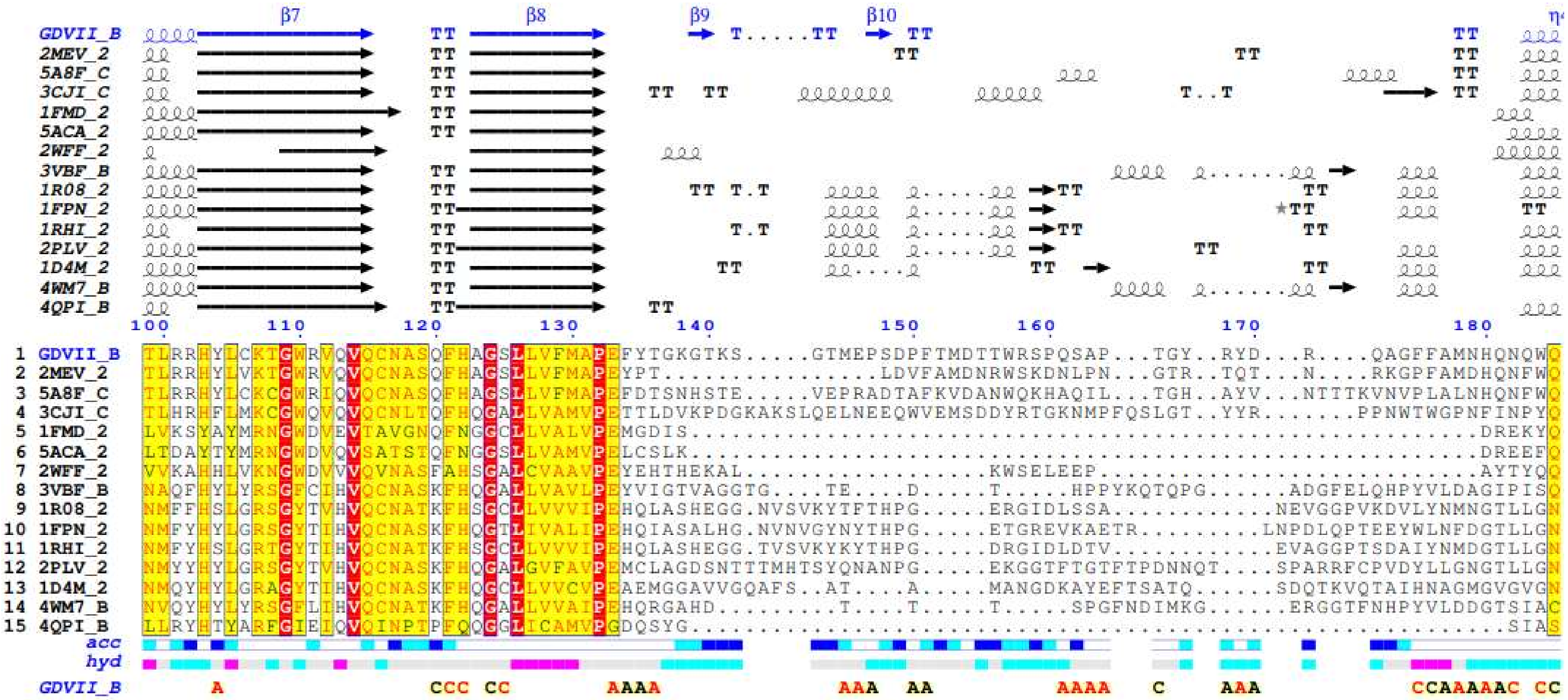

β7
β8
β9
β10
GDVII_B
2MEV_2
5A8F_C
3CJI_C
1FMD_2
5ACA_2
2WFF_2
3VBF_B
1R08_2
1FPN_2
1RHI_2
2PLV_2
1D4M_2
4WM7_B
4QPI_B
100
110
120
130
140
150
160
170
180
acc
hyd

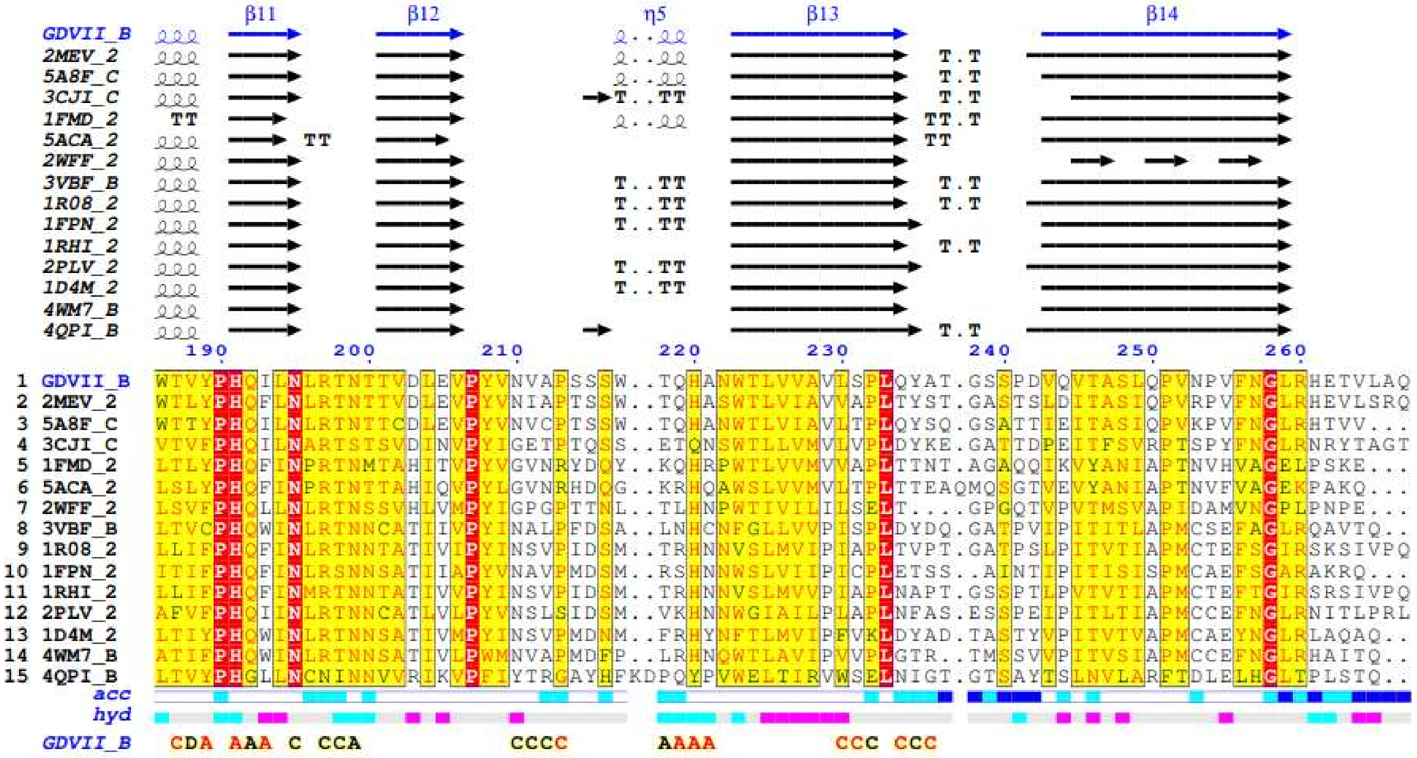

Figure S2. Sequence and structure-based alignment of TMEV VP2 with cardiovirus, senecavirus, aphthovirus and enterovirus representatives [1]. Chain B representing VP2 of the TMEV protomer was submitted to ENDscript2. A subset of structures provided by ENDscript2 was selected for the alignment and included: cardioviruses MEV and SAFV (PDB: 2MEV, 5A8F); senecavirus SVV-1 (PDB: 3CJI); aphthoviruses FMDV type C and SAT 2, ERAV (PDB: 1FMD, 5ACA, 2WFF); enteroviruses EV-71, HRV-B14, HRV-2, HRV-3, PV-1, CV-A9, EV-D68 (PDB: 3VBF, 1R08, 1FPN, 1RHI, 2PLV, 1D4M, 4WM7) and HAV (4QPI).

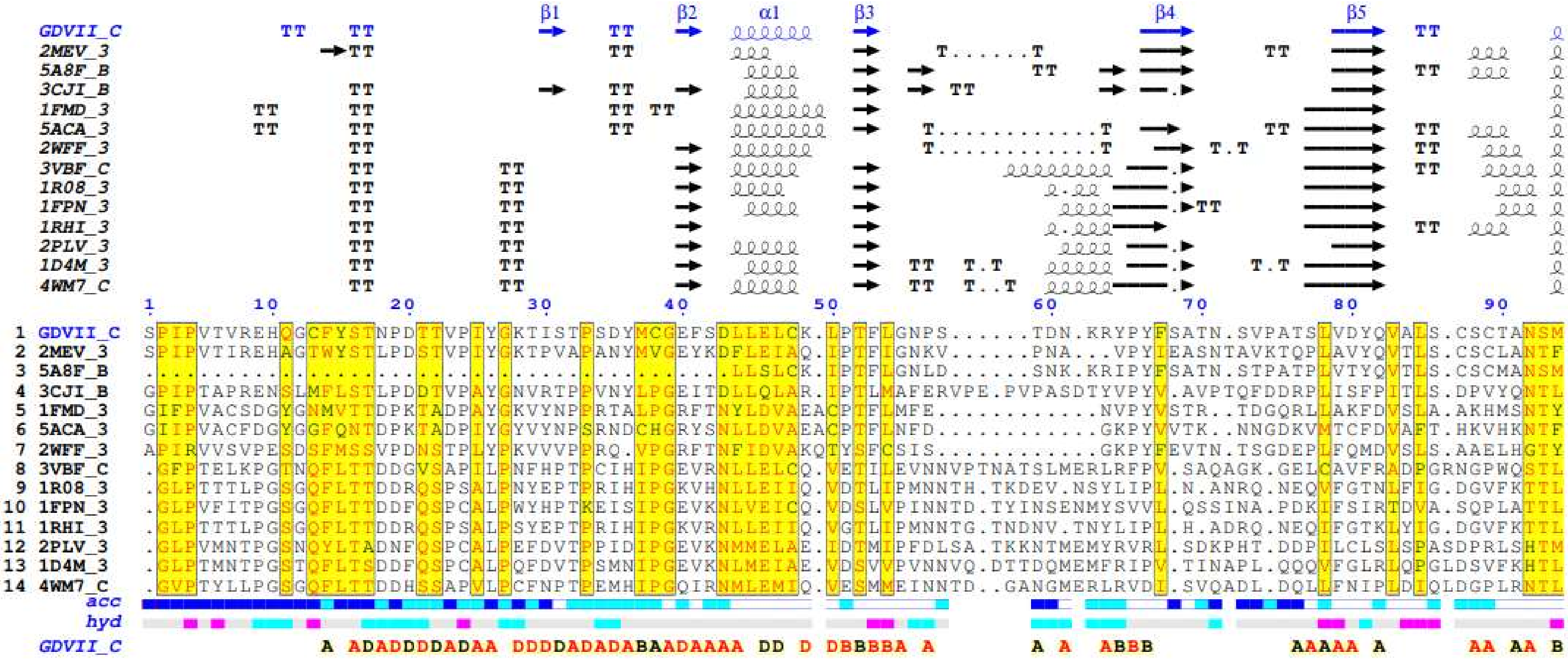
β1 β2 α1 β3 β4 β5
GDVII_C
2MEV_3
5A8F_B
3CJI_B
1FMD_3
5ACA_3
2WFF_3
3VBF_C
1R08_3
1FPN_3
1RHI_3
2PLV_3
1D4M_3
4WM7_C
1 10 20 30 40 50 60 70 80 90
1 GDVII_C SPIPVTVREHQGCFYSTNPDTTVPIYGKTISTPSDYMCGEFSDLLELCK.LPTFLGNPS......TDN.KRYPYFSATN.SVPATSLVDYQVALS.CSCTANSM
2 2MEV_3 SPIPVTIREHAGTWYSTLPDSTVPIYGKTPVAPANYMVGEYKDFLEIAQ.IPTFIGNKV......PNA...VPYIEASNTAVKTQPLAVYQVTLS.CSCLANTF
3 5A8F_B ....LLSLCK.IPTFLGNLD......SNK.KRIPYFSATN.STPATPLVTYQVTLS.CSCMANSM
4 3CJI_B GPIPTAPRENSLMFLSTLPDDTVPAYGNVRTPPVNYLPGEITDLLQLAR.IPTLMAFERVPE.PVPASDTYVPYV.AVPTQFDDRPLISFPITLS.DPVYQNTL
5 1FMD_3 GIFPVACSDGYGNMVTTDPKTADPAYGKVYNPPRTALPGRFTNYLDVAEACPTFLMFE...........NVPYVSTR..TDGQRLLAKFDVSLA.AKHMSNTY
6 5ACA_3 GIIPVACFDGYGGFQNTDPKTADPIYGYVYNPSRNDCHGRYSNLLDVAEACPTFLNFD...........GKPYVVTK..NNGDKVMTCFDVAFT.HKVHKNTF
7 2WFF_3 APIRVVSVPESDSFMSSVPDNSTPLYPKVVVPPRQ.VPGRFTNFIDVAKQTYSFCSIS............GKPYFEVTN.TSGDEPLFQMDVSLS.AAELHGTY
8 3VBF_C .GFPTELKPGTNQFLTTDDGVSAPILPNFHPTPCIHIPGEVRNLLELCQ.VETILEVNNVPTNATSLMERLRFPV.SAQAGK.GELCAVFRADPGRNGPWQSTL
9 1R08_3 .GLPTTTLPGSGQFLTTDDRQSPSALPNYEPTPRIHIPGKVHNLLEIIQ.VDTLIPMNNTH.TKDEV.NSYLIPL.N.ANRQ.NEQVFGTNLFIG.DGVFKTTL
10 1FPN_3 .GLPVFITPGSGQFLTTDDFQSPCALPWYHPTKEISIPGEVKNLVEICQ.VDSLVPINNTD.TYINSENMYSVVL.QSSINA.PDKIFSIRTDVA.SQPLATTL
11 1RHI_3 .GLPTTTLPGSGQFLTTDDRQSPSALPSYEPTPRIHIPGKVRNLLEIIQ.VGTLIPMNNTG.TNDNV.TNYLIPL.H.ADRQ.NEQIFGTKLYIG.DGVFKTTL
12 2PLV_3 .GLPVMNTPGSNQYLTADNFQSPCALPEFDVTPPIDIPGEVKNMMELAE.IDTMIPFDLSA.TKKNTMEMYRVRL.SDKPHT.DDPILCLSLSPASDPRLSHTM
13 1D4M_3 .GLPTMNTPGSTQFLTSDDFQSPCALPQFDVTPSMNIPGEVKNLMEIAE.VDSVVPVNNVQ.DTTDQMEMFRIPV.TINAPL.QQQVFGLRLQPGLDSVFKHTL
14 4WM7_C .GVPTYLLPGSGQFLTTDDHSSAPVLPCFNPTPEMHIPGQIRNMLEMIQ.VESMMEINNTD..GANGMERLRVDI.SVQADL.DQLLFNIPLDIQLDGPLRNTL
acc
hyd
GDVII_C A ADADDDDADAA DDDDADADABAADAAAA DD D DBBBBA A A A ABBB AAAAA A AA AA B

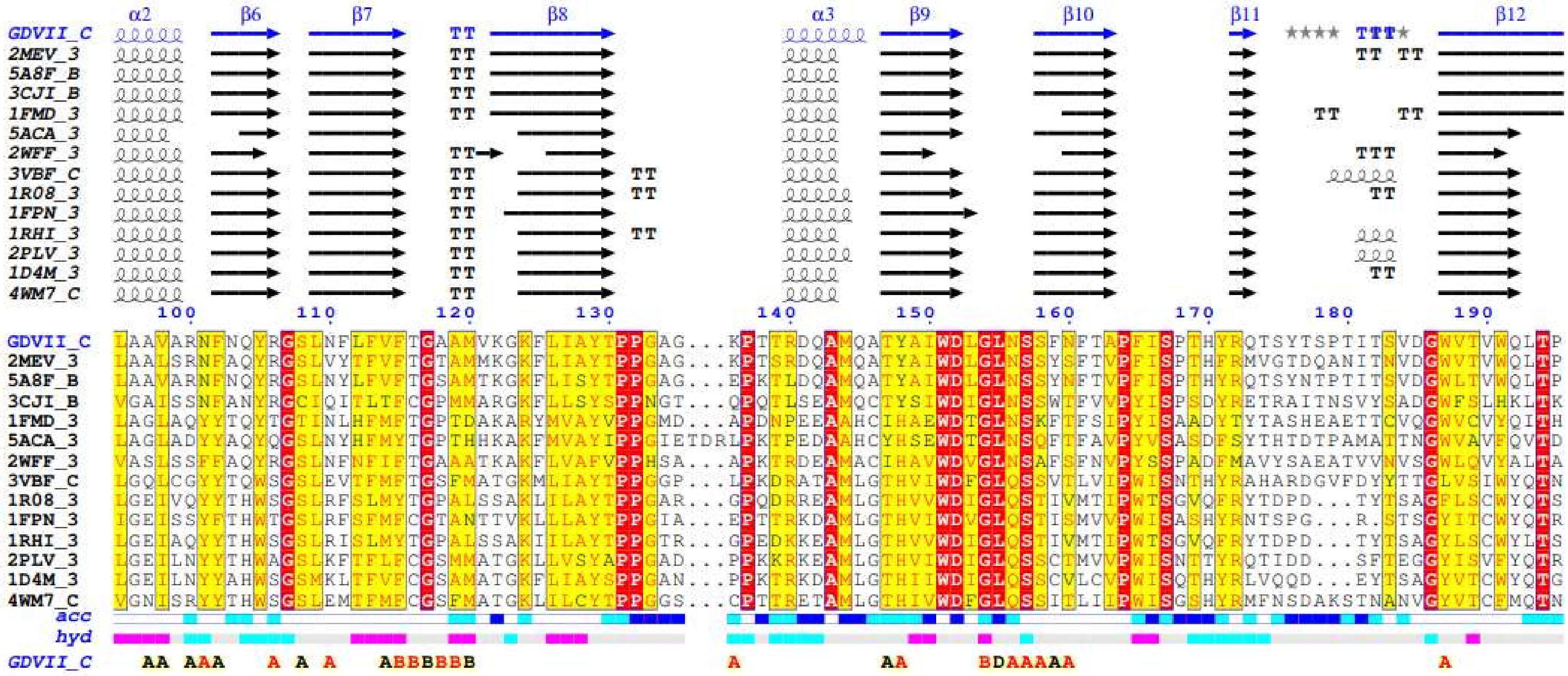
α2 β6 β7 β8 α3 β9 β10 β11 β12
GDVII_C
2MEV_3
5A8F_B
3CJI_B
1FMD_3
5ACA_3
2WFF_3
3VBF_C
1R08_3
1FPN_3
1RHI_3
2PLV_3
1D4M_3
4WM7_C
TT
TTT
100 110 120 130 140 150 160 170 180 190
1 GDVII_C LAAVARNFNQYRGSLNFLFVFTGAAMVKGKFLIAYTPPGAG...KPTTRDQAMQATYAIWDLGLNSSFNFTAPFISPTHYRQTSYTSPTITSVDGWVTVWQLTP
2 2MEV_3 LAALSRNFAQYRGSLVYTFVFTGTAMMKGKFLIAYTPPGAG...KPTSRDQAMQATYAIWDLGLNSSYSFTVPFISPTHFRMVGTDQANITNVDGWVTVWQLTP
3 5A8F_B LAAVARNFNQYRGSLNYLFVFTGSAMTKGKFLISYTPPGAG...EPKTLDQAMQATYAIWDLGLNSSYNFTVPFISPTHYRQTSYNTPTITSVDGWLTVWQLTP
4 3CJI_B VGAISSNFANYRGCIQITLTFCGPMMARGKFLLSYSPPNGT...QPQTLSEAMQCTYSIWDIGLNSSWTFVVPYISPSDYRETRAITNSVYSADGWFSLHKLTK
5 1FMD_3 LAGLAQYYTQYTGTINLHFMFTGPTDAKARYMVAYVPPGMD...APDNPEEAAHCIHAEWDTGLNSKFTFSIPYISAADYTYTASHEAETTCVQGWVCVYQITH
6 5ACA_3 LAGLADYYAQYQGSLNYHFMYTGPTHHKAKFMVAYIPPGIETDRLPKTPEDAAHCYHSEWDTGLNSQFTFAVPYVSASDFSYTHTDTPAMATTNGWVAVFQVTD
7 2WFF_3 VASLSSFFAQYRGSLNFNFIFTGAAATKAKFLVAFVPPHSA...APKTRDEAMACIHAVWDVGLNSAFSFNVPYSSPADFMAVYSAEATVVNVSGWLQVYALTA
8 3VBF_C LGQLCGYYTQWSGSLEVTFMFTGSFMATGKMLIAYTPPGGP...LPKDRATAMLGTHVIWDFGLQSSVTLVIPWISNTHYRAHARDGVFDYYTTGLVSIWYQTN
9 1R08_3 LGEIVQYYTHWSGSLRFSLMYTGPALSSAKLILAYTPPGAR...GPQDRREAMLGTHVVWDIGLQSTIVMTIPWTSGVQFRYTDPD...TYTSAGFLSCWYQTS
10 1FPN_3 IGEISSYFTHWTGSLRFSFMFCGTANTTVKLLLAYTPPGIA...EPTTRKDAMLGTHVIWDVGLQSTISMVVPWISASHYRNTSPG...R.STSGYITCWYQTR
11 1RHI_3 LGEIAQYYTHWSGSLRISLMYTGPALSSAKIILAYTPPGTR...GPEDKKEAMLGTHVVWDIGLQSTIVMTIPWTSGVQFRYTDPD...TYTSAGYLSCWYLTS
12 2PLV_3 LGEILNYYTHWAGSLKFTFLFCGSMMATGKLLVSYAPPGAD...PPKKRKEAMLGTHVIWDIGLQSSCTMVVPWISNTTYRQTIDD...SFTEGGYISVFYQTR
13 1D4M_3 LGEILNYYAHWSGSMKLTFVFCGSAMATGKFLIAYSPPGAN...PPKTRKDAMLGTHIIWDIGLQSSCVLCVPWISQTHYRLVQQD...EYTSAGYVTCWYQTG
14 4WM7_C VGNISRYYTHWSGSLEMTFMFCGSFMATGKLILCYTPPGGS...CPTTRETAMLGTHIVWDFGLQSSITLIIPWISGSHYRMFNSDAKSTNANVGYVTCFMQTN
acc
hyd
GDVII_C AA AAA A A A ABBBBBB A AA BDAAAAA A

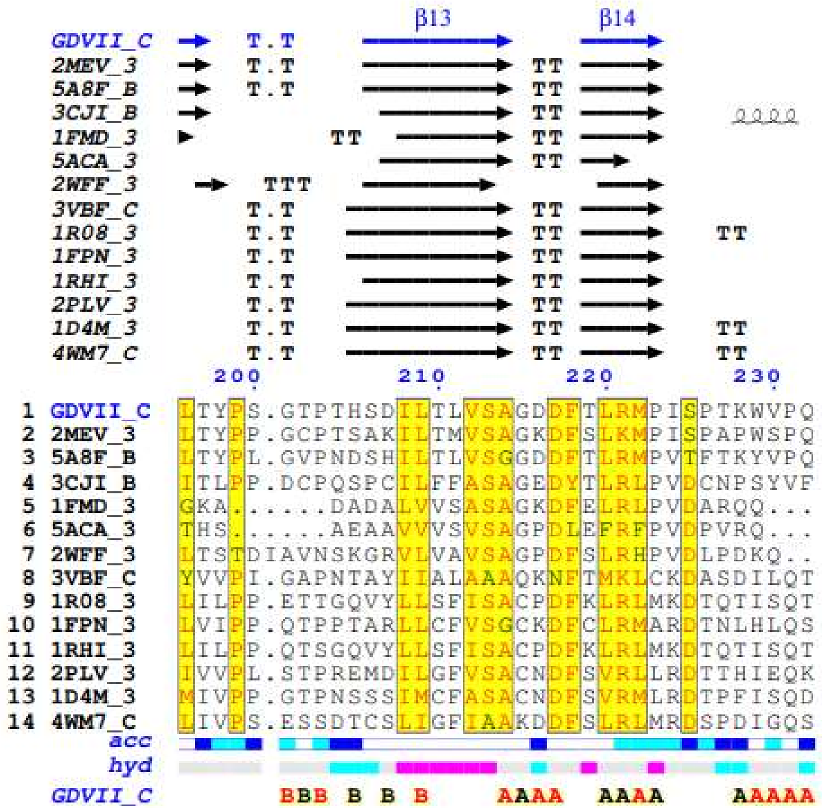

Figure S3. Sequence and structure-based alignment of TMEV VP3 with cardiovirus, senecavirus, aphthovirus and enterovirus representatives [1]. Chain C representing VP3 of the TMEV protomer was submitted to ENDscript2. A subset of structures provided by ENDscript2 was selected for the alignment and included: cardioviruses MEV and SAFV (PDB: 2MEV, 5A8F); senecavirus SVV-1 (PDB: 3CJI); aphthoviruses FMDV type C and SAT 2, ERAV (PDB: 1FMD, 5ACA, 2WFF); and enteroviruses EV-71, HRV-B14, HRV-2, HRV-3, PV-1, CV-A9, EV-D68 (PDB: 3VBF, 1R08, 1FPN, 1RHI, 2PLV, 1D4M, 4WM7).

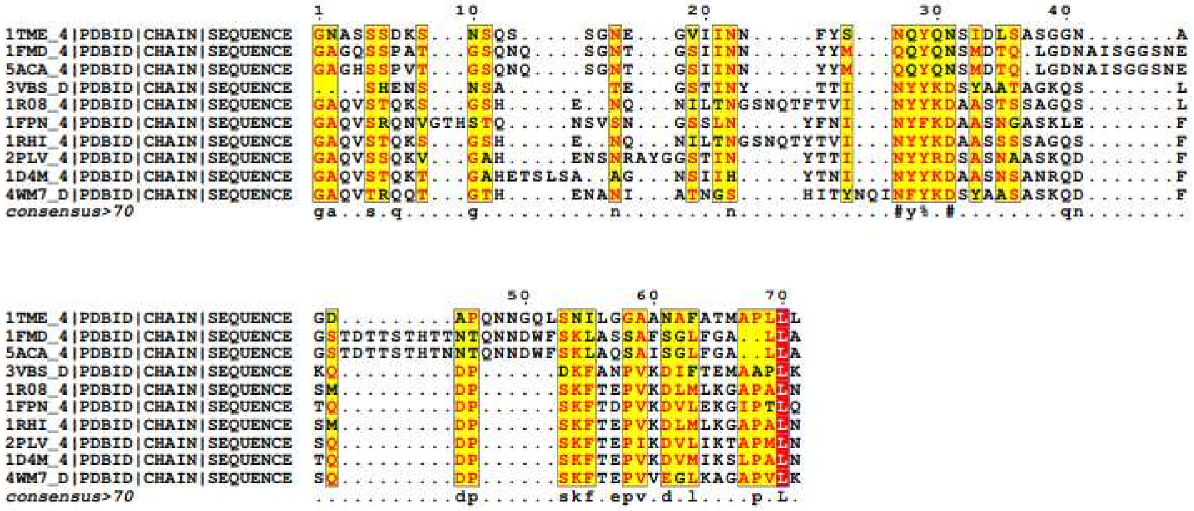

Figure S4. Sequence-based alignment of TMEV, aphthovirus, cardiovirus and enterovirus VP4 proteins [1]. VP4 protein sequences were obtained from the PDB database: TMEV (PDB: 1TME); aphthoviruses FMDV type C and SAT 2, ERAV (PDB: 1FMD, 5ACA, 2WFF); and enteroviruses EV-71, HRV-B14, HRV-2, HRV-3, PV-1, CV-A9, EV-D68 (PDB: 3VBS, 1R08, 1FPN, 1RHI, 2PLV, 1D4M, 4WM7) and were submitted to ESpript (built into ENDscript) to produce a multiple sequence alignment of the amino acid sequences.

Reference:

1. Upfold, N.S. Exploring the structural integrity of a picornavirus capsid, Rhodes University, 2019. http://hdl.handle.net/10962/131837.
